# Efficient Plastic Waste Recycling to Value‐Added Products by Integrated Biomass Processing

**DOI:** 10.1002/cssc.201902880

**Published:** 2020-01-08

**Authors:** Kassem Beydoun, Jürgen Klankermayer

**Affiliations:** ^1^ Institut für Technische und Makromolekulare Chemie RWTH Aachen University Worringerweg 2 52074 Aachen Germany

**Keywords:** acetals, circular economy, polymers, recycling, waste valorization

## Abstract

The industrial production of polymeric materials is continuously increasing, but sustainable concepts directing towards a circular economy remain rather elusive. The present investigation focuses on the recycling of polyoxymethylene polymers, facilitated through combined catalytic processing of polymer waste and biomass‐derived diols. The integrated concept enables the production of value‐added cyclic acetals, which can flexibly function as solvents, fuel additives, pharmaceutical intermediates, and even monomeric materials for polymerization reactions. Based on this approach, an open‐loop recycling of these waste materials can be envisaged in which the carbon content of the polymer waste is efficiently utilized as a C1 building block, paving the way to unprecedented possibilities within a circular economy of polyoxymethylene polymers.

## Graphical abstract

**Plastics reborn**: A flexible open‐loop recycling of polyoxymethylene polymer waste materials is established in which the carbon content of the polymer waste is efficiently utilized as a C1 building block for the synthesis of value‐added cyclic acetals for use as solvents, fuel additives, intermediates, and monomers, paving the way to unprecedented possibilities within a circular economy of polyoxymethylene polymers.

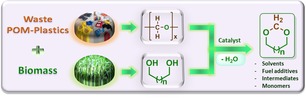

Most plastics are made of synthetic polymers that are produced by the repetitive linkage of versatile small monomers. The continuous optimization of manufacture processes and tailoring of material properties has paved the way to mass production of a broad diversity of plastics. Thus, highly versatile consumer products with low weight, high strength, and extreme durability are broadly available at low costs. Consequently, plastics have become crucial in a variety of strategic industrial sectors and represent essential materials for construction, transportation, and packaging. The production of plastic materials is steadily growing and since 1950 around 8300 million tons of this materials have been synthesized.^1^ As a result, worldwide generation of plastic waste has dramatically increased and is currently around 150 million tons per year. However, 79 % of this plastic waste ends up in landfills, resulting in up to 2.41 million tons of robust waste entering our environment every year.^2^ Consequently, the reuse or substitution of the plastic materials is currently strongly fostered, as efficient and sustainable recycling strategies remain rather elusive.^3^ Moreover, available recycling concepts are not cost competitive and produce polymer materials of lower quality, hampering the development of a circular economy. Therefore, the development of effective recycling processes within a circular economy approach is of utmost importance, ideally producing not only monomers for new plastics, but value‐added products or intermediates for other supply chains.^4^ Herein, a sustainable concept is introduced, enabling efficient plastic recycling through combined catalytic processing with biomass‐derived chemicals, yielding high‐value platform chemicals.

The present investigation focuses on the recycling of polyoxymethylene polymers (POM, also known as polyacetal), a thermoplastic material produced by the homo‐ or copolymerization of formaldehyde in a production capacity of around 1.7 Mtons per year.^5^ POM plastics have been known for more than 40 years and are used in precision parts requiring high stiffness, low friction, and excellent dimensional stability in automotive interiors.^6^ Currently, POM can be repurposed via injection molding processes, which are limited by material degradation and release of formaldehyde. Chemical recycling has not been intensively investigated and few approaches focusing on the transformation of POM into formaldehyde or trioxane have been reported.^7^ In this study, we have demonstrated effective recycling of POM plastic materials, which is facilitated by the combined catalytic processing of polymer waste and biomass‐derived diols. In detail, POM reacts with either biomass‐derived^8^ or recycled4c glycols to produce chemical products such as 1,3‐dioxolane, 1,3‐dioxane, or 1,3‐dioxepane (Scheme 1). These useful cyclic acetal products can serve as solvents, fuel additives, pharmaceutical intermediates, and even monomeric materials for polymerization reactions.^9^

**Scheme 1:**
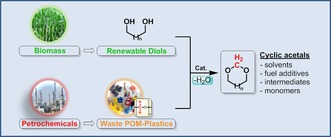
Efficient approach for the upcycling of polyoxymethylene plastics in combination with biomass‐derived diols to versatile cyclic acetal products.

The investigation was initiated with a catalytic reaction of granules of commercial POM homopolymer together with 1,3‐propanediol. The biomass‐derived substrate, 1,3‐propanediol, can be industrially obtained via fermentation processes using the renewable resources corn syrup or glycerol.^10^ For the first reactions, 1,4‐dioxane was chosen as solvent and a variety of Brønsted acid catalysts were screened, promoting the depolymerization of POM to the formaldehyde monomer and fostering the subsequent condensation to the cyclic acetal (Table 1). In the absence of any acid, the envisaged formation of 1,3‐dioxane product was not observed and the starting polymer remained undissolved in the reaction mixture (Table 1, entry 1). Gratifyingly, with hydrochloric acid as catalyst the POM polymer started to dissolve and 1,3‐dioxane product was formed in 31 % yield after 2 h (Table 1, entry 2), corroborating the viability of the envisaged cascade transformation.

**Table 1:** Acid‐catalyzed transformation of POM with 1,3‐propanediol towards the synthesis of 1,3‐dioxane.^[a]^ 
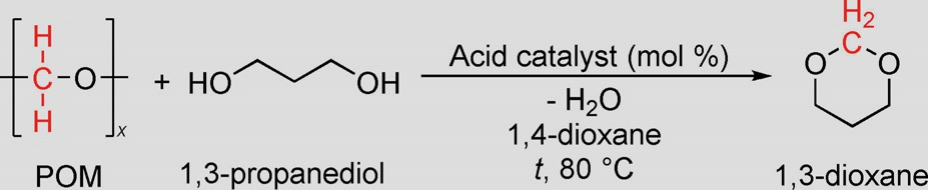

Entry	Catalyst	*x* [mol %]	Diol/POM [equiv]	*t* [h]	Yield [%]
1	no acid	–	3	2	0
2	HCl	12	3	2	31
3	*p‐*TsOH	5	3	2	1
4	HNTf_2_	5	3	2	20
5	TfOH	5	3	2	44
6	TfOH	5	3	8	99
7	Sc(OTf)_3_	5	3	2	6
8	Al(OTf)_3_	5	3	2	10
9	Bi(OTf)_3_	5	3	2	41
10	Bi(OTf)_3_	5	3	8	99
11	Bi(OTf)_3_	5	1.2	2	96
12^[b]^	Bi(OTf)_3_	5	1.2	2	99

[a] POM (51–54 mg, 1.7–1.8 mmol), 1,3‐propanediol (1.2–3 equiv), acid catalyst (*x* mol %), 1,4‐dioxane (2 mL), 80 °C, 2–8 h. [b] 2.5 mL of 1,4‐dioxane. Yields were determined by ^1^H NMR spectroscopy using mesitylene as an internal standard.

When *p*‐toluenesulfonic acid (*p*‐TsOH) was tested as catalyst, only trace amount of the 1,3‐dioxane product could be detected, whereas the stronger acid trifluoromethanesulfonimide (HNTf_2_) afforded the cyclic acetal in 20 % yield (Table 1, entries 3 and 4). However, with triflic acid (TfOH), 1,3‐dioxane was obtained in 44 % yield after 2 h and 99 % yield after 8 h of reaction time (Table 1, entries 5 and 6). From these initial results, the effectiveness of Brønsted acids for the catalytic depolymerization of the POM polymer and subsequent condensation with 1,3‐propanediol could be demonstrated. In the next development step, metal triflates with bifunctional Brønsted and Lewis behavior were investigated as catalysts.^11^ After screening several Lewis acids (Table 1, entries 7 and 8; see also the Supporting Information), Bi(OTf)_3_ catalyst demonstrated superior activity and afforded the 1,3‐dioxane product in 41 % yield after 2 h, and 99 % yield after 8 h (Table 1, entries 9 and 10). The comparable reactivity observed with Bi(OTf)_3_ and TfOH could be attributed to partially hidden Brønsted acid properties of the Lewis acid upon partial alcoholysis (by 1,3‐propandiol) or hydrolysis (by water byproduct). Thus, the dissociation of Bi(OTf)_3_ Lewis acid unlocks the hidden Brønsted acidity, while maintaining the Lewis acidic behavior. Moreover, the Lewis acidity of Bi(OTf)_3_ can additionally promote activation of the hydroxy groups of the diol substrate. In this manner, the formation of a Bi‐metallacycle intermediate has been previously reported to facilitate the condensation of glycols.^11^ Interestingly, when only 1.2 equivalents of 1,3‐propandiol were used, the product could be obtained in up to 96 % yield in only 2 h (Table 1, entry 11). With additional 1,4‐dioxane, the solvation of the polymeric substrate could be facilitated, resulting in 99 % yield of the 1,3‐dioxane product (Table 1, entry 12).

We then focused on optimization of the solvation–depolymerization process by studying the influence of temperature on the possible increase of the reactivity in presence of 1,3‐propanediol (Table 2). As expected, the dissolving/depolymerization of the starting POM polymer slowed down at lower temperatures of 60 and 70 °C, affording the desired product in 5 and 26 % yield compared to 96 % yield at 80 °C (Table 2, entries 1–3). On increasing the reaction temperature to 100 °C, the POM polymer granules were completely dissolved in the solution after only 20 min and the 1,3‐dioxane product was obtained in 97 % yield, compared to only 12 % yield at 80 °C (Table 2, entries 4 and 5). This higher reactivity at 100 °C temperature led to the study of the effect of lower catalyst loadings. When the reaction time was extended to 40 min, the starting material was completely converted and the desired product was obtained in 99 % yield at low catalyst loading of 1 mol % (Table 2, entry 6). Interestingly, when the amount of starting substrate was increased fourfold, we obtained the 1,3‐dioxane product in 95 % yield at a low catalyst loading of 1 mol % and in just 90 min of reaction time (Table 2, entry 7).

**Table 2:** Bi(OTf)_3_‐catalyzed depolymerization–condensation of POM with 1,3‐propanediol for the synthesis of 1,3‐dioxane under different reaction conditions.^[a]^ 
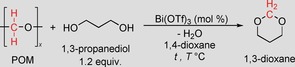

Entry	*x* [mol %	*T* [°C]	*t*	Yield [%]
1	5	60	2 h	5
2	5	70	2 h	26
3	5	80	2 h	96
4	5	80	20 min	12
5	5	100	20 min	97
6	1	100	40 min	99
7^[b]^	1	100	90 min	95

[a] POM (51–54 mg, 1.7–1.8 mmol), 1,3‐propanediol (1.2 equiv), Bi(OTf)_3_ (*x* mol %), 1,4‐dioxane (2 mL). [b] POM (210 mg, 7 mmol) was used. Yields were determined by ^1^H NMR spectroscopy using mesitylene as an internal standard.

In the subsequent investigation, ^1^H NMR experiments should provide information on the rates of depolymerization and condensation within the cascade transformation. Thus, we carried out a study on the time profile of the selective synthesis of 1,3‐dioxane from POM polymer/1,3‐propanediol mixtures by using Bi(OTf)_3_ as catalyst (Figure 1; see also the Supporting Information). The reaction revealed a constant rate of formation of 1,3‐dioxane in the first 100 min, indicating immediate 1,3‐dioxane formation after POM depolymerization. However, after 100 min the reaction slows down until the remaining POM substrate is fully converted, finally reaching 99 % yield. This indicates that the reactivity of the reaction is largely dominated by the rate of solvation–depolymerization of the POM polymer granules.

**Figure 1:**
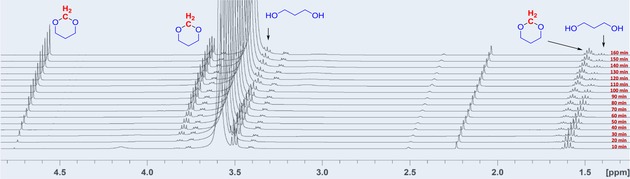
^1^H‐NMR spectra of the reaction of POM with 1,3‐propanediol to afford 1,3‐dioxane at different time intervals (see the Supporting Information for details).

Based on optimized conditions with 1,3‐propanediol, we next evaluated the scope of this reaction with structural variations on the biomass‐derived diols.8a–8h In the first set of experiments 1,2‐butanediol, 1,2‐pentanediol, and 1,2‐hexanediol were used as substrates together with POM polymer. The corresponding cyclic acetal products were obtained in excellent yield of 93–98 % (Table 3, entries 1–3). Furthermore, when sterically constrained 2,3‐butanediol and 2,4‐pentanediol were used, lower yields of 65 % and 57 % were achieved (Table 3, entries 4 and 5). Interestingly, the use of 1,3‐butanediol as substrate resulted in the formation of 4‐methyl‐1,3‐dioxane acetal in an excellent yield of 98 % (Table 3, entry 6). Subsequently, we attempted the challenging synthesis of seven‐membered cyclic acetals starting from biomass‐derived 1,4‐diols. When 1,4‐butanediol and 1,4‐pentanediol were employed as substrates, the corresponding seven‐membered cyclic acetal products (1,3‐dioxepanes) were formed in 26 % and 59 % yield (Table 3, entries 7 and 8) in addition to the formation of a set of different linear glycolic acetals as by‐products (see Supporting Information). The higher reactivity of 1,4‐pentanediol in comparison to 1,4‐butanediol can be attributed to the presence of a secondary alcohol functionality, improving the cyclization–acetalization step towards a more stable seven‐membered‐ring acetal product.

**Table 3:** Bi(OTf)_3_‐catalyzed synthesis of cyclic acetals using variable diols and POM polymer as substrates.^[a]^ 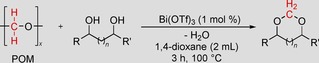

Entry	Diol substrate	Cyclic acetal product	Yield [%]
1	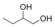	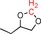	93
2	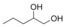	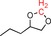	97
3	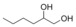	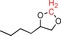	98
4	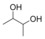	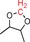	65
5	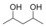	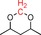	57
6	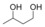	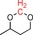	98
7	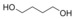	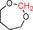	26
8	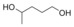	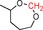	59

[a] POM (210 mg, 7 mmol), diol (1.2 equiv), Bi(OTf)_3_ (46 mg, 1 mol %), 1,4‐dioxane (2 mL), 100 °C, 3 h. Yields and selectivity were determined by ^1^H NMR spectroscopy using mesitylene as an internal standard.

Grounded on the principles of green chemistry and previous investigations from our group on polymer recycling, a reaction without solvent use was targeted.^12^ Thus, a transformation only with the application of the starting materials 1,3‐propanediol and POM polymer was performed. In the initial phase of the reaction the absence of adapted solvent reduces the solvation and subsequent depolymerization rates of POM. However, the accumulation of the produced 1,3‐dioxane product in the reaction mixture continuously enhances the POM solvation step in a self‐breeding (autosolvation) manner. More specifically, starting with neat conditions, the reaction resulted in the formation of 1,3‐dioxane with an overall yield of 86 % with only 0.2 mol % of acid catalyst (Scheme 2). More interestingly, when the self‐breeding approach is supported with an initial addition of 1,3‐dioxane product (0.5 mL), the solvation–depolymerization of POM was enhanced and the yield of the produced 1,3‐dioxane increased to 93 % (Scheme 2). This important finding strongly facilitates the development of an adapted process concept, as final purification only requires the distillative separation of water and the cyclic acetal product.

**Scheme 2:**
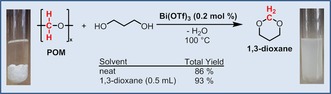
Synthesis of 1,3‐dioxane in a solvent‐free approach using very low catalyst loadings.

In addition to the biomass‐derived diols, a set of these diols can also be derived from the recycling of polymeric plastic waste.4c, 8a, 8e Two specific examples of the latter case are represented by ethylene glycol and propylene glycol, which can be obtained from waste polyethylene terephthalate (PET) and polylactic acid (PLA).4c Moreover, the expected cyclic acetal products of these glycol substrates have already been introduced (mainly 1,3‐dioxolane) as commercial solvents for polar polymers, in paint stripping formulations, and as a general clean‐up solvent for epoxy and urethane systems, as well as for the synthesis of selected pharmaceutical intermediates.^13^ Remarkably, when ethylene glycol and propylene glycol were employed in the reaction with POM substrate, the corresponding cyclic acetal products, 1,3‐dioxolane and 4‐methyl‐1,3‐dioxolane, were obtained in 66 % and 93 % yield, respectively (Scheme 3), clearly validating the versatility and substrate flexibility of the developed concept.

**Scheme 3:**
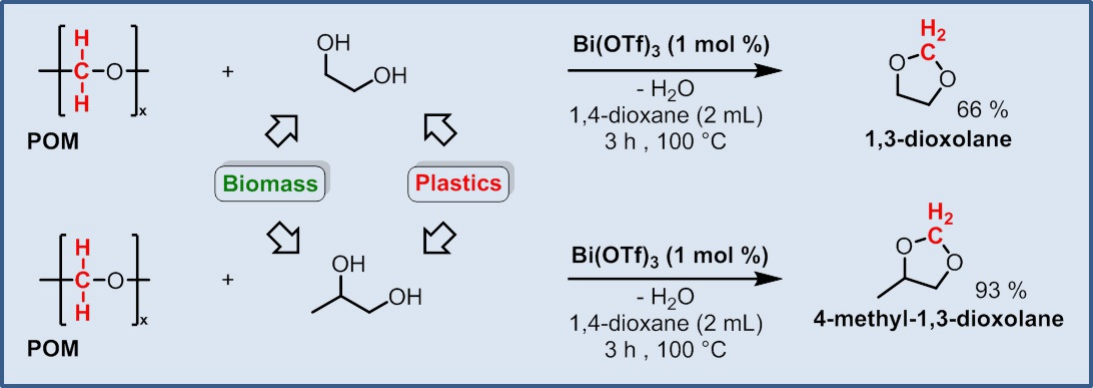
Synthesis of 1,3‐dioxolane or 4‐methyl‐1,3‐dioxolane starting from POM polymer in the presence of ethylene or propylene glycol.

In the last decade, market demand for POM polymer has doubled, leading to an increase of the production capacity to 1.7 million tons per annum in 2015. POM polymers are widely used in the production of a variety of commercial plastic products, including consumer goods such as toys, zippers, clips, cosmetic containers, and pens, which are typical items found in contaminated shore and sea areas. Moreover, POM has also been used for complicated engineering applications, mainly in the automotive industry for production of around 3000 different components used for external & internal auto parts. Consequently, in a final set of experiments, our approach of utilizing POM polymer as an alternative C1 source for the synthesis of cyclic acetals was applied on some commercial consumer products, such as small gears, baggage clips, and disposable lighters, as well as old laboratory joint clips (Figure 2). These commercial POM plastic wastes were shredded to small fragments (<3 mm in size) and treated under the developed conditions with 1,3‐propanediol. Astonishingly, the waste material selectively forms the envisaged 1,3‐dioxane products in very good yield (Figure 2 and Table S10 in the Supporting Information). Interestingly, the POM polymer content of these plastics was fully converted, whereas the dyes and additives could be precipitated and removed by filtration. More specifically, 1,3‐dioxane was formed in 87, 90, 88, 92, and 88 % yield, starting from baggage clips, joint clips, disposable lighters, and plastic gears, respectively, using only 0.2 mol % of Bi(OTf)_3_ catalyst.

**Figure 2:**
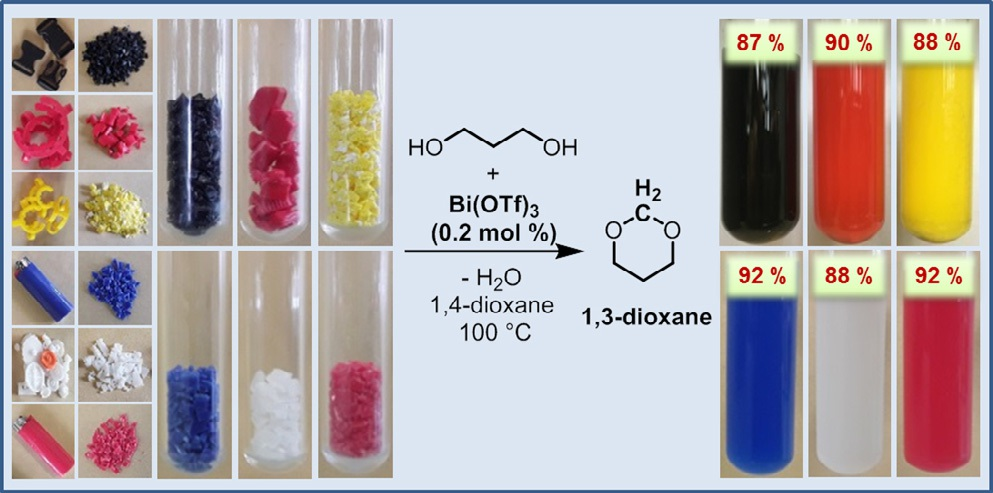
Upcycling of POM plastic wastes as a C1 building block in the Bi(OTf)_3_‐catalyzed synthesis of 1,3‐dioxane by using 1,3‐propanediol.

Finally, a scale‐up of the reaction starting from approximately 6 g of a mixture of commercial POM waste under neat conditions was performed. After product distillation, 91 % yield of pure 1,3‐dioxane product could be isolated. The detailed steps of the straightforward recycling concept on a larger scale are shown in Figure 3, clearly corroborating the potential of the facile approach. Moreover, the performance of the reaction under neat conditions and the ease of separation of the product disclose the future potential of this method.

**Figure 3:**
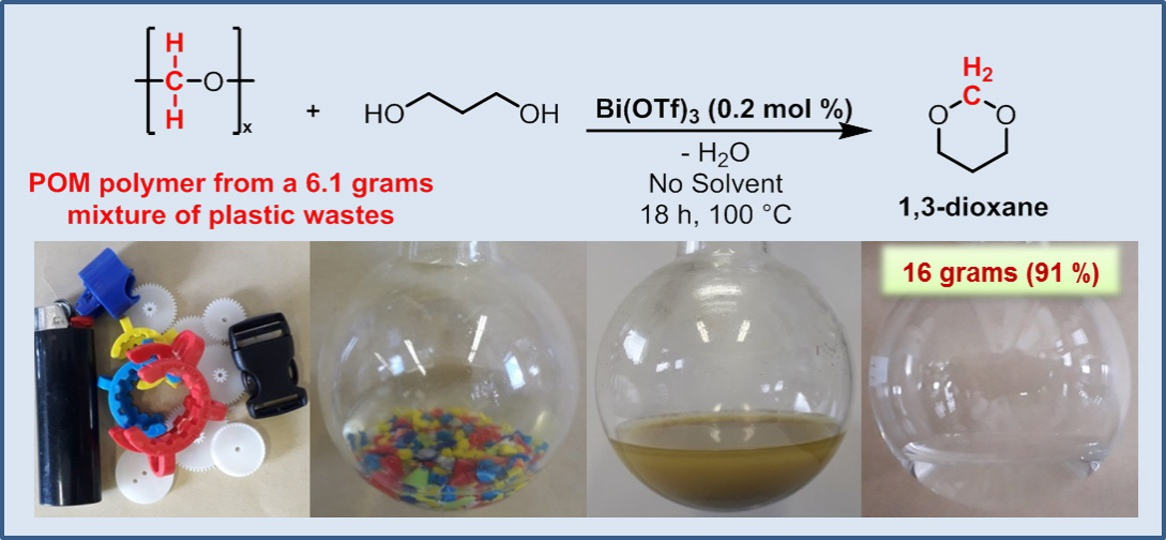
Reaction of commercial POM polymer from a mixture of plastic wastes and 1,3‐propanediol followed by the distillation of 1,3‐dioxane product.

The efficiency of this recycling approach of POM plastic waste can be evaluated by the converted carbon content of the used plastic waste. Interestingly, the complete carbon content of the POM homopolymer is efficiently converted into the acetal unit of the cyclic product driven by the excellent yields obtained for these products. Moreover, the addressed recycling method of POM is associated with a very low E‐factor,^14^ where water is produced as the only byproduct of the acetalization reaction.

In the present work, the development of a sustainable concept for effective polymer recycling was targeted, focusing on the transformation of polyoxymethylene waste materials. The basic concept was grounded on a combined catalytic processing of polymer waste material and biomass derived diols. The respective integrated concept enabled a selective cascade reaction, encompassing effective depolymerization and condensation, leading to cyclic acetals. The robustness of this approach allowed the flexible formation of various cyclic products in high yield by facile modification of biomass‐derived diol. Based on this approach, an efficient open‐loop recycling of these waste materials can be envisaged, paving the way to unprecedented possibilities within a circular economy of polyoxymethylene plastic polymers.

## Experimental Section

### General procedure for the synthesis of 1,3‐dioxane from homo‐POM plastic polymer and 1,3‐propanediol

All experiments were conducted in 5 mL sealable heavy‐walled glass vials equipped with a magnetic stir bar. After weighing Bi(OTf)_3_ (0.058 g, 0.088 mmol), homo‐POM (51–54 mg, 1.7–1.8 mmol; calculated based on the formaldehyde H_2_CO repetition unit), and diol (0.156 g, 2.05 mmol) in the vial, 1,4‐dioxane (2 mL) was added and the vial was sealed with a 20 mm aluminum seal equipped with septa. The reaction mixture was stirred and heated to 100 °C by using a customized aluminum heating block. After 2 h, the vial was cooled to room temperature and NMR samples were prepared by using [D_6_]DMSO solvent and mesitylene as internal standard.

## Conflict of interest

*The authors declare no conflict of interest*.

## Supporting information

As a service to our authors and readers, this journal provides supporting information supplied by the authors. Such materials are peer reviewed and may be re‐organized for online delivery, but are not copy‐edited or typeset. Technical support issues arising from supporting information (other than missing files) should be addressed to the authors.

Supplementary
