# Supplementary material for: Efficient Plastic Waste Recycling to Value‐Added Products by Integrated Biomass Processing
Source: ChemSusChem. 2020 Jan 8;13(3):488–92. doi: 10.1002/cssc.201902880 (PMC7027741; doi:10.1002/cssc.201902880)
Supplement: Supplementary file 1 — Supplementary [file CSSC-13-488-s001.pdf]

CHEMISTRY & SUSTAINABILITY
CHEMSUSCHEM
ENERGY & MATERIALS

Supporting Information

Efficient Plastic Waste Recycling to Value-Added Products by Integrated Biomass Processing

Kassem Beydoun and Jürgen Klankermayer*[a]

cssc_201902880_sm_miscellaneous_information.pdf

This file includes:

General Procedure

Optimization of reaction conditions for the synthesis of 1,3-dioxane using POM

Study of the reaction scope using variable diols

Upcycling of commercial POM-plastic wastes for the synthesis of 1,3-dioxane using 1,3-propanediol

NMR-Spectra of the synthesis of cyclic acetals from diols and POM

General Procedure

All experiments were conducted in 5 mL sealable heavy-walled glass vials equipped with a magnetic stir bar. After weighing $Bi(OTf)_3$ (0.058 g, 0.088 mmol), homo-POM (51-54 mg, equivalent to 1.7-1.8 mmol of $-CH_2O-$), and diol (0.156 g, 2.05 mmol) in the vial, 2 mL of 1,4-dioxane solvent is added and the vial was sealed with a 20 mm aluminum seal equipped with Septa. The reaction mixture was stirred and heated to 100 °C using a customized aluminum heating block. After 2 h the vial was cooled to room temperature and NMR samples were prepared using mesitylene as internal standard. All reagents were commercially available and used as received, unless stated otherwise. ^{1}H, and ^{13}C spectra were recorded with spectrometer Bruker AV400 at room temperature in DMSO-*d6*. Chemical shifts are given in ppm relative to DMSO-*d6* (^{1}H: δ = 2.5 ppm and ^{13}C: δ = 39.0 ppm).

The POM used for the optimization of reaction conditions is an Acetal-Homopolymer POMH (brand names Derlin/Tenac) which is typically a high molecular weight homopolymer of formaldehyde repeating unit capped by acetyl end groups. As the molecular weight of the POMH polymer is very high (Av. 15,000 – 100,000),[1] the masses weighed were calculated based on molecular weight of the formaldehyde ($-H_2CO-$) repeating unit representing the major content of

the polymer (acetyl end caps represent < 1 % mass content). Some operating properties of POMH are summarized in the table below.

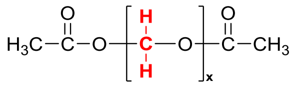

Polyacetal Homopolymer POMH with acetyl end groups

Properties of POM homopolymer			
**Chemical Resistance**		**Electrical Properties**	
Acids - concentrated	Poor	Dielectric constant @1MHz	3.7
Acids - dilute	Poor - Fair	Dielectric strength (kV mm^{-1})	20
Alcohols	Good-Fair	Dissipation factor @ 1MHz	0.005
Alkalis	Poor	Surface resistivity (Ohm/sq)	10^{15}
Aromatic hydrocarbons	Good	Volume resistivity (Ohmcm)	10^{15}
Greases and Oils	Good	**Thermal Properties**	
Halogenated Hydrocarbons	Good-Poor	Coefficient of thermal expansion ($x10^{-6}$ K^{-1})	122
Halogens	Poor	Heat-deflection temperature - 0.45MPa (°C)	170
Ketones	Good - Fair	Heat-deflection temperature - 1.8MPa (°C)	135
**Physical Properties**		Specific heat (J K^{-1} kg^{-1})	1500
Density (g cm^{-3})	1.42	Thermal conductivity @23 °C (W m^{-1} K^{-1})	0.22-0.24
Flammability	HB	Upper working temperature (°C)	80-120
Limiting oxygen index (%)	15	**Mechanical Properties**	
Radiation resistance	Poor	Coefficient of friction	0.2 - 0.35
Resistance to Ultra-violet	Poor	Elongation at break (%)	40 - 75
Water absorption - equilibrium (%)	0.6 - 0.9	Hardness - Rockwell	M94
Water absorption - over 24 hours (%)	0.25	Izod impact strength (J m^{-1})	75 - 130
		Poisson's ratio	0.35
		Tensile modulus (GPa)	2.9 - 3.1
		Tensile strength (MPa)	70

Optimization of reaction conditions for the synthesis of 1,3-dioxane using POM

Table S1. Acid variation for the acid-catalyzed synthesis of 1,3-dioxane using POM homopolymer and 1,3-propandiol.[a]

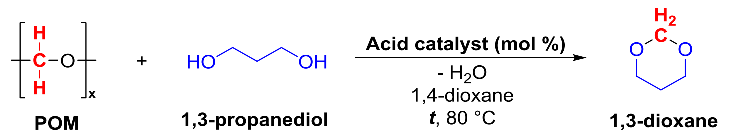

Entry	Cat.	mol%	*t* [h]	Yield [%]
1	no acid	--	2	0
2	HCl	12	2	31
3	*p*-TsOH	5	2	1
4	$HNTf_2$	5	2	20
5	TfOH	5	2	44
6	TfOH	5	8	99
7	$B(C_6F_5)_3$	5	2	0
8	LiOTf	5	2	0
9	NaOTf	5	2	0
10	$Mg(OTf)_2$	5	2	0
11	$Zn(OTf)_2$	5	2	0
12	$Sn(OTf)_2$	5	2	29
13	$Y(OTf)_3$	5	2	0
14	$Sc(OTf)_3$	5	2	6
15	$Al(OTf)_3$	5	2	10
16	$Bi(OTf)_3$	5	2	41
17	$Bi(OTf)_3$	5	8	99
18[b]	Amberlyst-15	-	18	0
19	Nafion-NR50	4.2	18	0

[a] POM (51-54 mg, 1.7-1.8 mmol), 1,3-propanediol (3 equiv), Acid catalyst (mol%), 1,4-dioxane (2 mL), 80 °C, 2-8 h. Yields were determined by ^{1}H-NMR spectroscopy using mesitylene as an internal standard; [b] 51 mg of Amberlyst-15 used.

Table S2. Optimizing diol/POM loading ratio for the $Bi(OTf)_3$-catalyzed synthesis of 1,3-dioxane using POM homopolymer and 1,3-propandiol.[a]

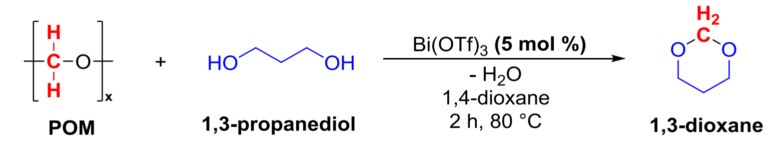

Entry	Diol/POM [equiv.]	Yield [%]
1	0.4	93
2	0.6	94
3	0.8	94
4	1.0	93
5	1.2	96
6	1.4	87
7	2	62
8	3	41

[a] POM (51-54 mg, 1.7-1.8 mmol), 1,3-propanediol (0.4-3 equiv), $Bi(OTf)_3$ (5 mol%), 1,4-dioxane (2 mL), 80 °C, 2 h. Yields were determined by ^1H-NMR spectroscopy using mesitylene as an internal standard.

Table S3. Variation of acid loading for the $Bi(OTf)_3$-catalyzed synthesis of 1,3-dioxane using POM homopolymer and 1,3-propandiol.[a]

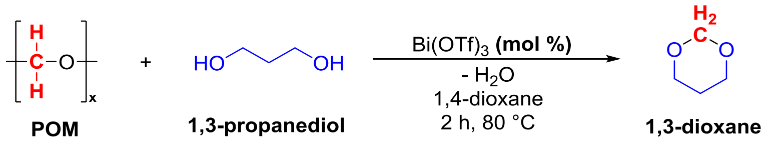

Entry	mol%	Yield [%]
1	1	51
2	2	73
3	3	81
4	4	90
5	5	96

[a] POM (51-54 mg, 1.7-1.8 mmol), 1,3-propanediol (1.2 equiv), $Bi(OTf)_3$ catalyst (1-5 mol%), 1,4-dioxane (2 mL), 80 °C, 2 h. Yields were determined by ^1H-NMR spectroscopy using mesitylene as an internal standard.

Table S4. Time-profile of the $Bi(OTf)_3$-catalyzed synthesis of 1,3-dioxane using POM homopolymer and 1,3-propandiol.[a]

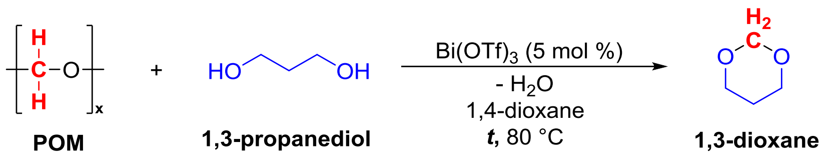

Entry	*t* [min]	Yield [%]
1	10	4
2	20	12
3	30	19
4	40	22
5	50	34
6	60	39
7	70	51
8	80	62
9	90	75
10	100	80
11	110	89
12	120	96
13	130	96
14	140	97
15	150	97
16	160	99

[a] POM (51-54 mg, 1.7-1.8 mmol), 1,3-propanediol (1.2 equiv), $Bi(OTf)_3$ (5 mol%), 1,4-dioxane (2 mL), 80 °C, ***t*** min. Yields were determined by ^{1}H-NMR spectroscopy using mesitylene as an internal standard.

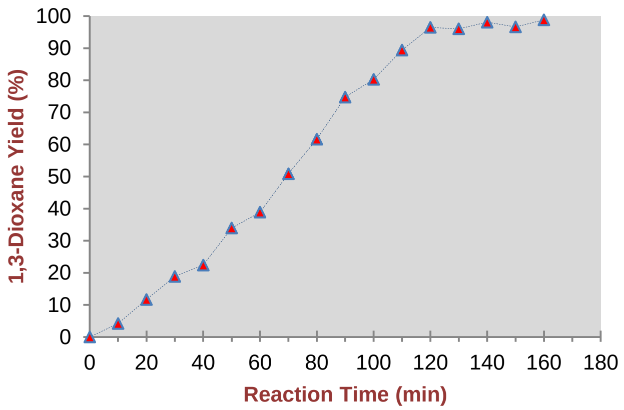

Fig. S1. Time-profile of the reaction of POM polymer with 1,3-propanediol showing the yields of 1,3-dioxane Product *vs* reaction time.

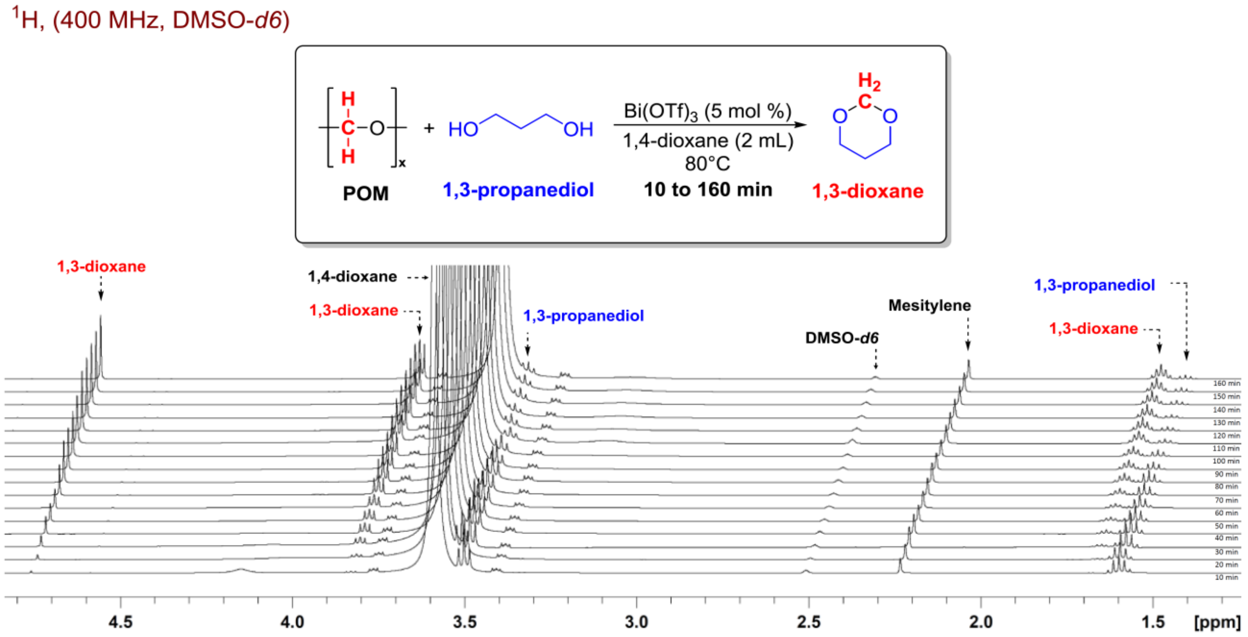

Fig. S2. ^{1}H-NMR spectra (400 MHz) vs reaction time.

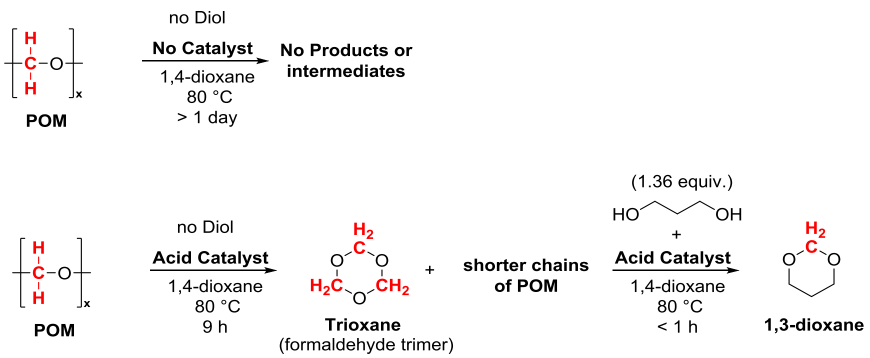

Scheme S1. Control experiments to determine possible reaction intermediates.

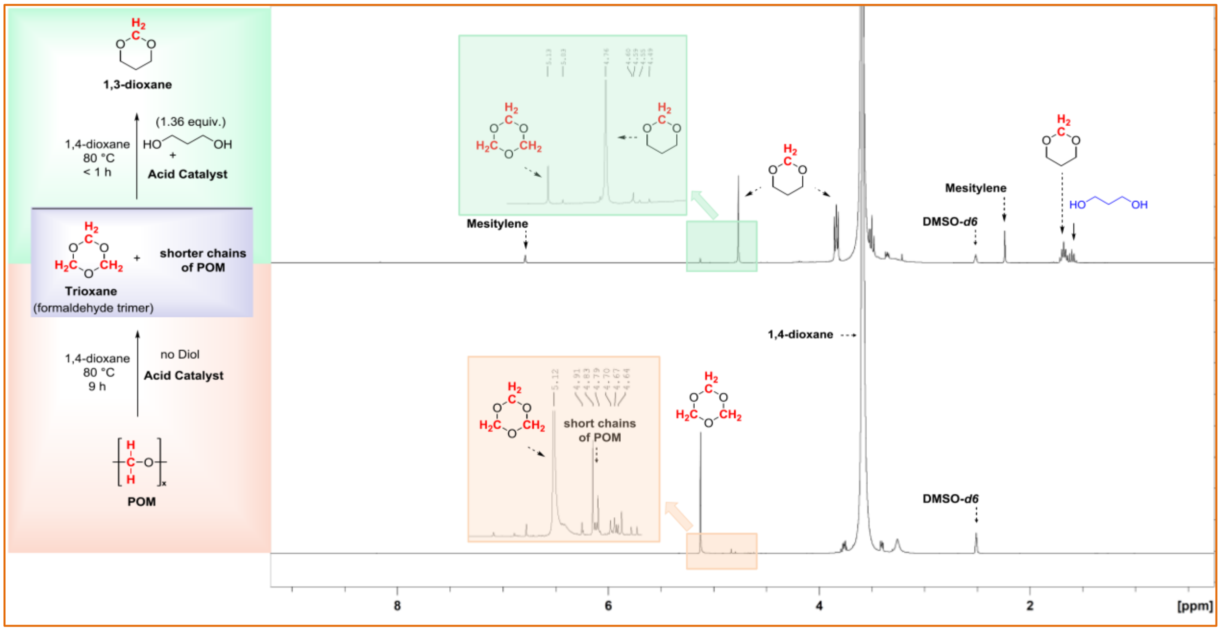

Scheme S2. ^{1}H-NMR spectra (400 MHz) of the control experiments showing possible reaction intermediates.

Table S5. Solvation-effect of the Bi(OTf)$_3$-catalyzed synthesis of 1,3-dioxane using POM homopolymer and 1,3-propandiol.[a]

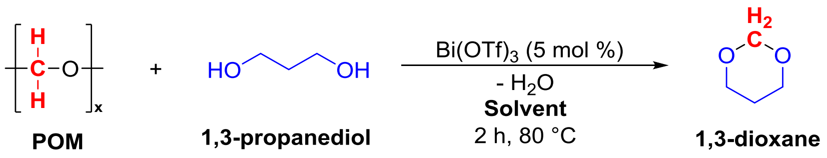

Entry	Solvent [mL]	Yield [%]
1	neat	7
2	0.5	65
3	1	79
4	1.5	90
5	2	96
6	2.5	99

[a] POM (51-54 mg, 1.7-1.8 mmol), 1,3-propanediol (1.2 equiv), Bi(OTf)$_3$ (5 mol%), 1,4-dioxane (mL), 80 °C, 2 h; [b] at 100 °C. Yields were determined by ^{1}H-NMR spectroscopy using mesitylene as an internal standard.

Table S6. Optimization of reaction temperature for the Bi(OTf)$_3$-catalyzed synthesis of 1,3-dioxane using POM homopolymer and 1,3-propandiol.[a]

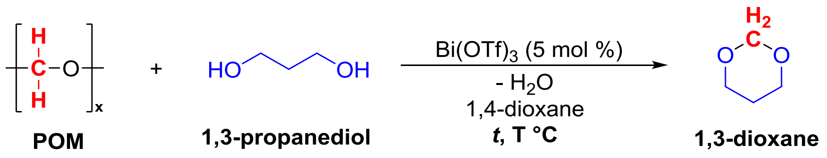

Entry	T [ºC]	*t* [min]	Yield [%]
1	60	120	5
2	70	120	26
3	80	20	12
4	80	40	22
5	80	120	96
6	100	20	97
7	100	40	97

[a] POM (51-54 mg, 1.7-1.8 mmol), 1,3-propanediol (1.2 equiv), Bi(OTf)$_3$ (5 mol%), 1,4-dioxane (2 mL), **T** °C, ***t*** min. Yields were determined by ^{1}H-NMR spectroscopy using mesitylene as an internal standard.

Table S7. General optimization for the Bi(OTf)$_3$-catalyzed synthesis of 1,3-dioxane using POM homopolymer and 1,3-propandiol at 100 ºC.[a]

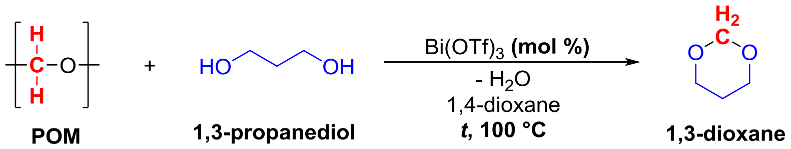

Entry	POM [mmol]	Bi(OTf)$_3$ [mol%]	*t* [min]	Yield [%]
1	1.8	1	20	60
2	1.8	2	20	83
3	1.8	3	20	95
4	1.8	4	20	96
5	1.8	5	20	97
6	1.8	1	40	99
7	1.8	2	40	99
8	1.8	3	40	98
9	1.8	4	40	97
10	1.8	5	40	97
11	7	1	90	95
12	35	0.2	840	90

[a] POM (x mmol), 1,3-propanediol (1.2 equiv), Bi(OTf)$_3$ (mol%), 1,4-dioxane (2 mL), 100 °C, ***t*** min. Yields were determined by ^{1}H-NMR spectroscopy using mesitylene as an internal standard.

Table S8. Study of Bi(OTf)$_3$ catalyst reactivity employing solvent-free conditions and reloading cycles of POM/1,3-propandiol substrates.[a]

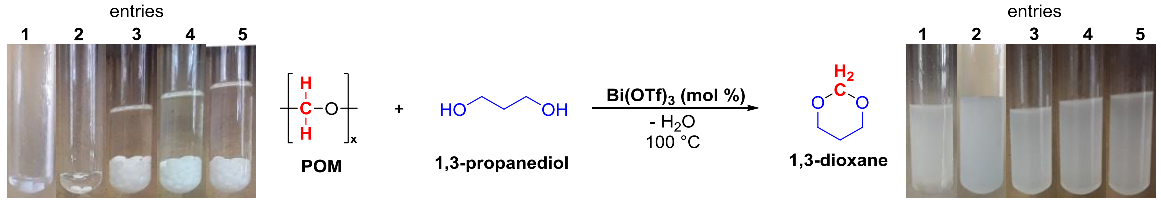

Entry	Solvent	Bi(OTf)$_3$ [µmol]		POM [mmol]	Overall mol% of Bi(OTf)$_3$	Overall POM/Bi(OTf)$_3$	*t* [h]	Overall Yield [%]
1	neat	90	Initial run	1.8	5	20	2	73
			1st reloading cycle	1.8	2.5	40	6	78
			2nd reloading cycle	3.6	1.25	80	6	82
			3rd reloading cycle	6.6	0.65	150	6	n.d
			4th reloading cycle	7.5	0.42	236	6	n.d
			5th reloading cycle	14	0.25	392	6	80
			**Overall loads**	**35.2**	**0.25**	**392**	**32**	**80**
2	1,3-dioxane (0.25 mL)	90	Initial run	1.8	5	20	2	n.d
			1st reloading cycle	5.15	1.3	77	6	79
			2nd reloading cycle	7.16	0.63	157	6	77
			3rd reloading cycle	9.31	0.38	260	6	n.d
			4th reloading cycle	14.11	0.24	417	6	n.d
			5th reloading cycle	14.11	0.17	575	6	91
			**Overall loads**	**51.7**	**0.17**	**575**	**32**	**91**
3	neat	75	One run	**35.2**	**0.21**	**470**	**16**	**86**
4	1,3-dioxane (0.5 mL)	75	One run	**35**	**0.21**	**466**	**16**	**93**
5	1,3-dioxane (1 mL)	75	One run	**35**	**0.21**	**466**	**16**	**91**

[a] POM (x mmol), 1,3-propanediol (1.2 equiv), Bi(OTf)$_3$ (mol%), 100 °C, *t* min; n.d: not determined; Yields were determined by ^{1}H-NMR spectroscopy using mesitylene as an internal standard.

Study of the reaction scope using variable diols

Table S9. $Bi(OTf)_3$-catalyzed synthesis of cyclic acetals using POM polymer and variable diols as substrates.[a]

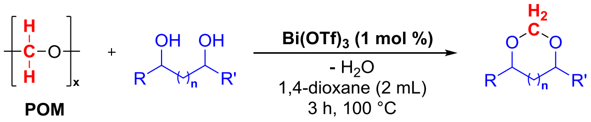

Entry	Diol	Cyclic Product	Yield [%]
1	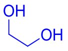	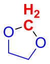	66
2	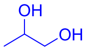	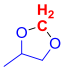	93
3	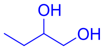	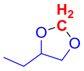	93
4	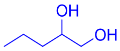	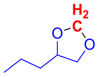	97
5	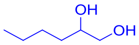	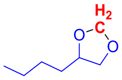	98
6	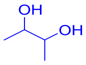	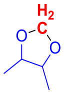	65
7	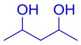	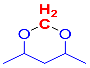	57
8	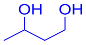	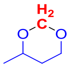	98
9	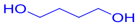	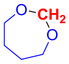	26
10	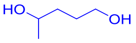	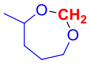	59

[a] POM (210 mg, 7 mmol), diol (1.2 equiv), $Bi(OTf)_3$ (46 mg, 1 mol%), 1,4-dioxane (2 mL), 100 °C, 3 h; Yields and selectivity were determined by ^1H-NMR spectroscopy using mesitylene as an internal standard.

Section 4. Upcycling of commercial POM-plastic wastes via the synthesis of 1,3-dioxane using 1,3-propanediol

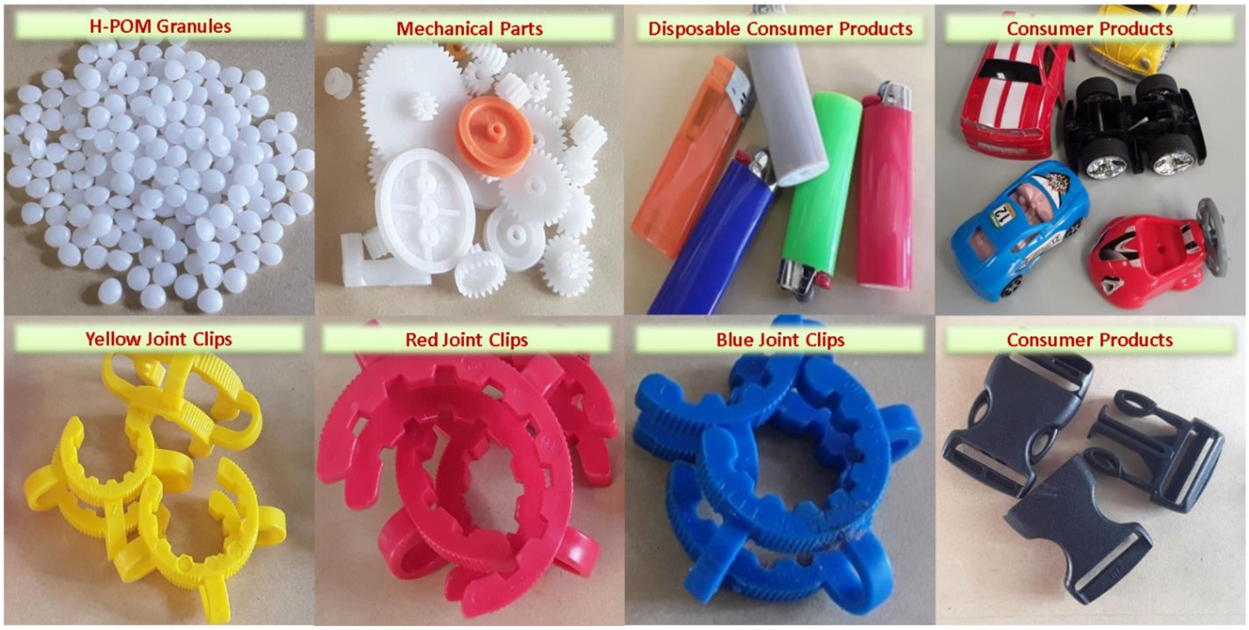

Fig. S3. Commercially polyxymethyelene (POM) plastic products used in this work.

Table S10. Bi(OTf)$_3$-catalyzed upcycling of POM-plastic wastes from commercial sources using 1,3-propanediol to synthesis 1,3-dioxane.[a]

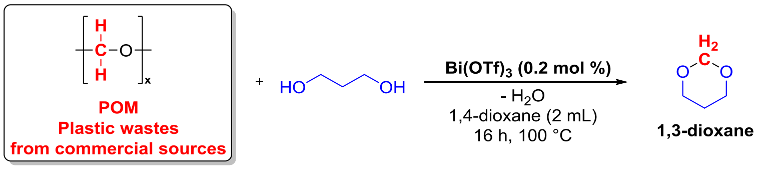

Entry	Commercial waste polymer	Reaction mixture before/after precipitation of additives	Filtrate	Product Yield [%]
1	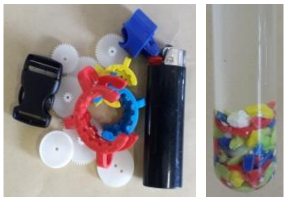	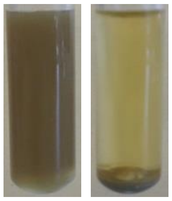	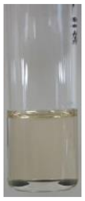	89
2	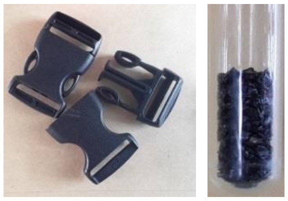	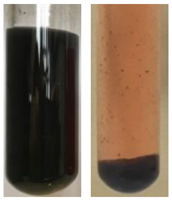	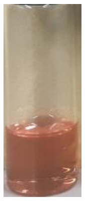	87

3	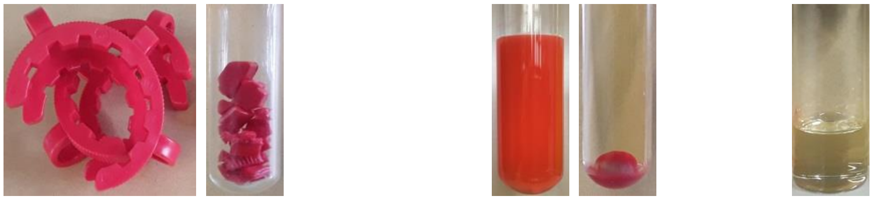	90
4	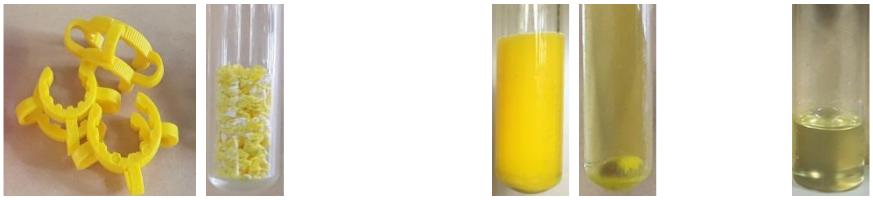	88
5	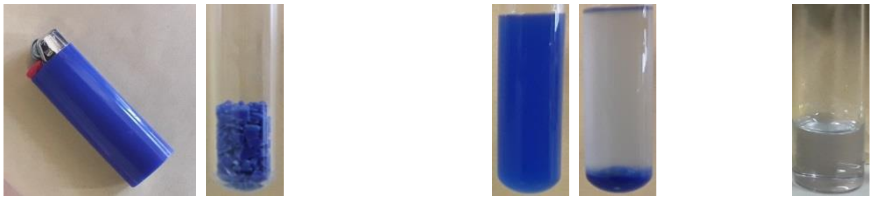	92
6	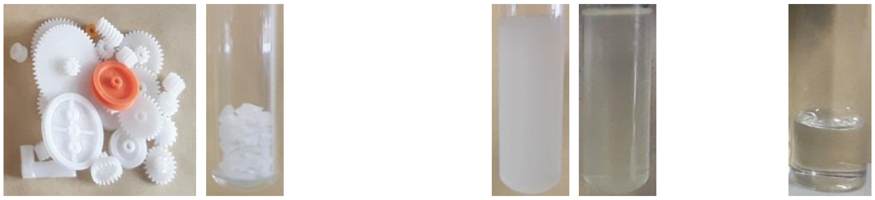	88
7	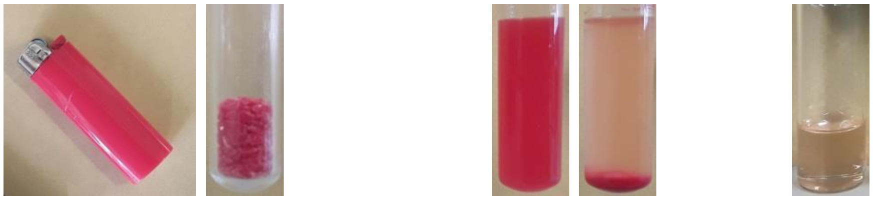	92
8	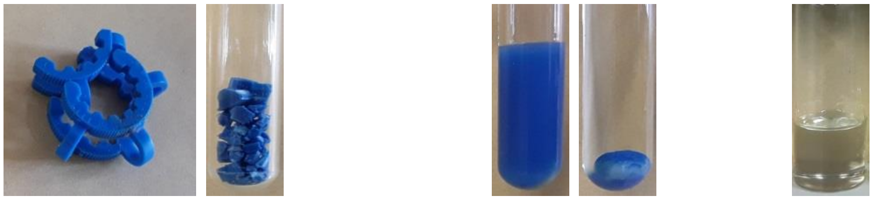	91
9	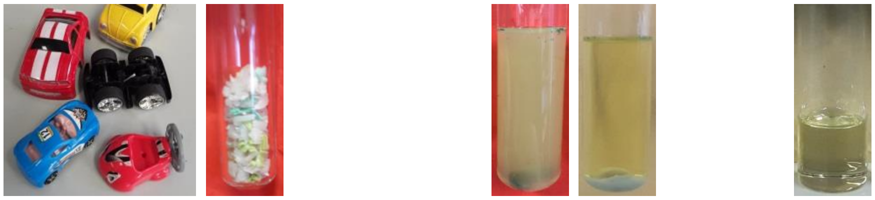	85
10	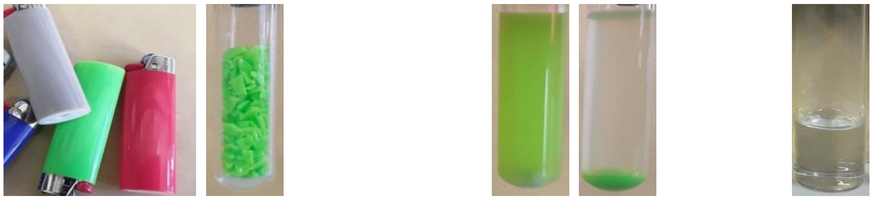	93

11	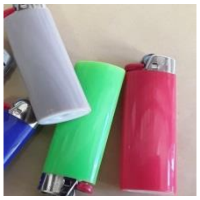 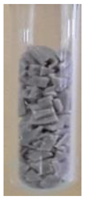	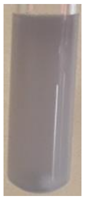 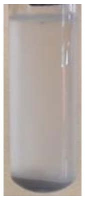	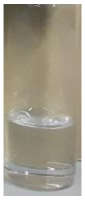	93

[a] POM (1050 mg, 35 mmol), diol (1.2 equiv), $Bi(OTf)_3$ (46 mg, 0.2 mol%), 1,4-dioxane (2 mL), 100 °C, 16 h; Yields and selectivity were determined by ^{1}H-NMR spectroscopy using mesitylene as an internal standard.

NMR-Spectra of the synthesis of cyclic acetals and polyoxymethylene POM under optimized conditions

NMR-Spectra of the synthesis of 1,3-dioxane from 1,3-Propandiol and polyoxymethylene POM under optimized conditions.

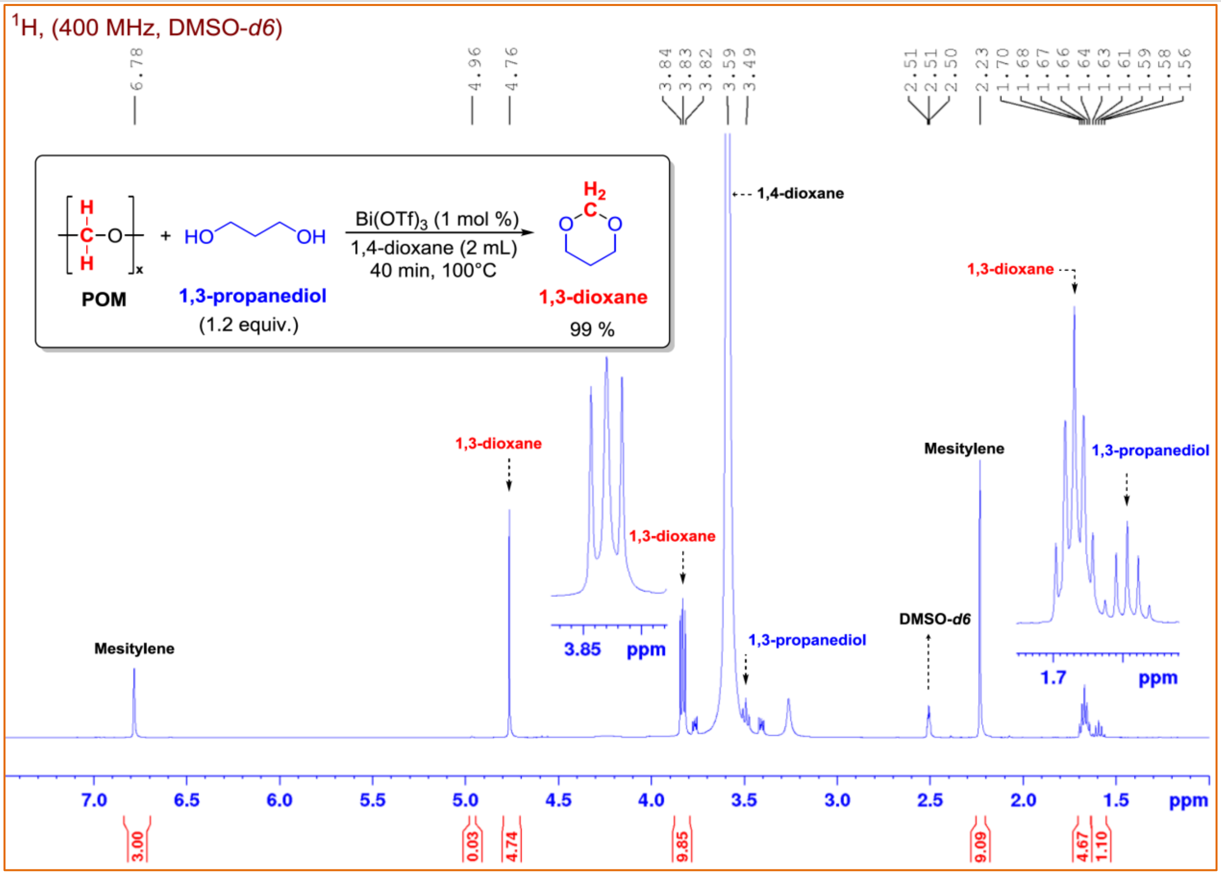

Fig. S4. ^{1}H-NMR spectrum (400 MHz) of the crude 1,4-dioxane reaction mixture of the acetalization of 1,3-propanediol using Polyoxymethyelene homopolymer POM.

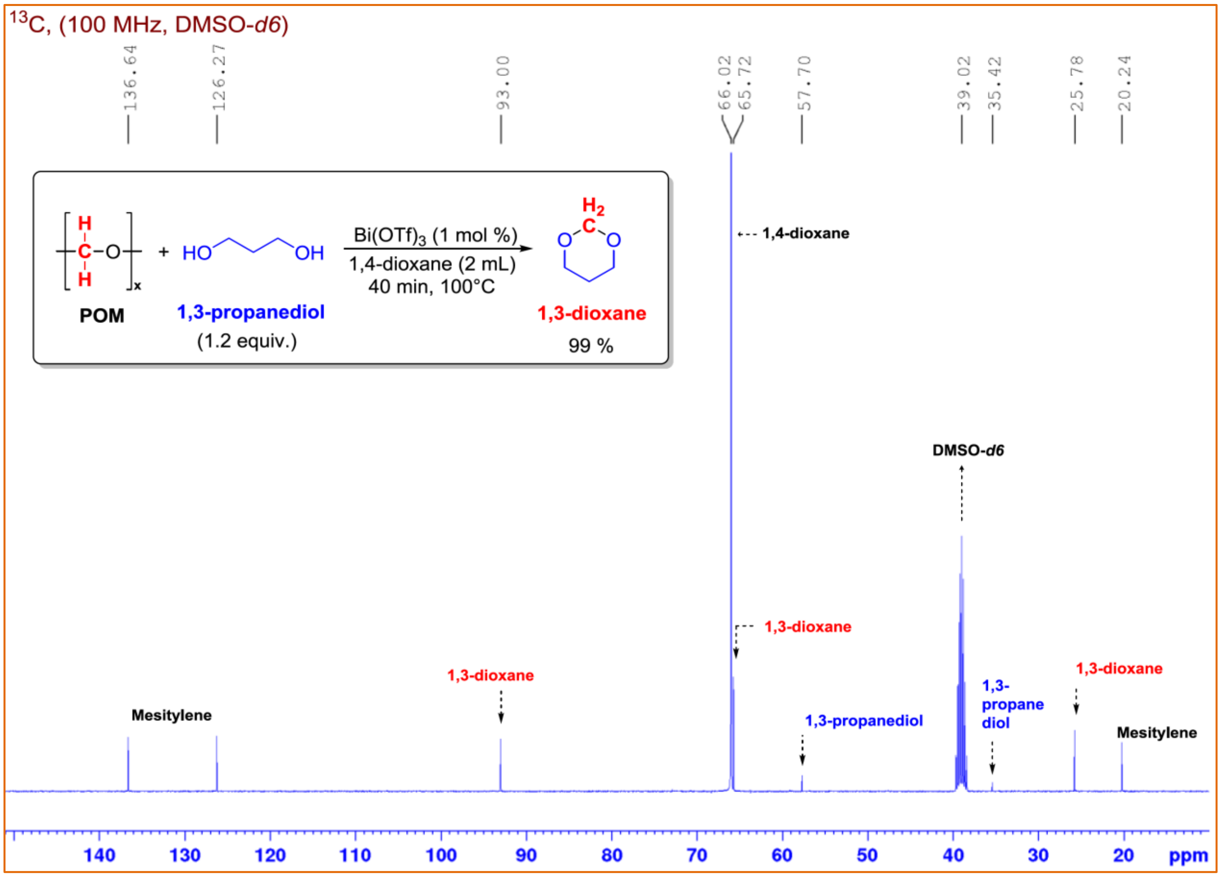

Fig. S5. ^{13}**C-NMR spectrum (100 MHz) of the crude 1,4-dioxane reaction mixture of the acetalization of 1,3-propanediol using Polyoxymethyelene homopolymer POM.**

NMR-Spectra of the synthesis of 1,3-dioxane from 1,3-Propandiol and polyoxymethylene POM under neat conditions.

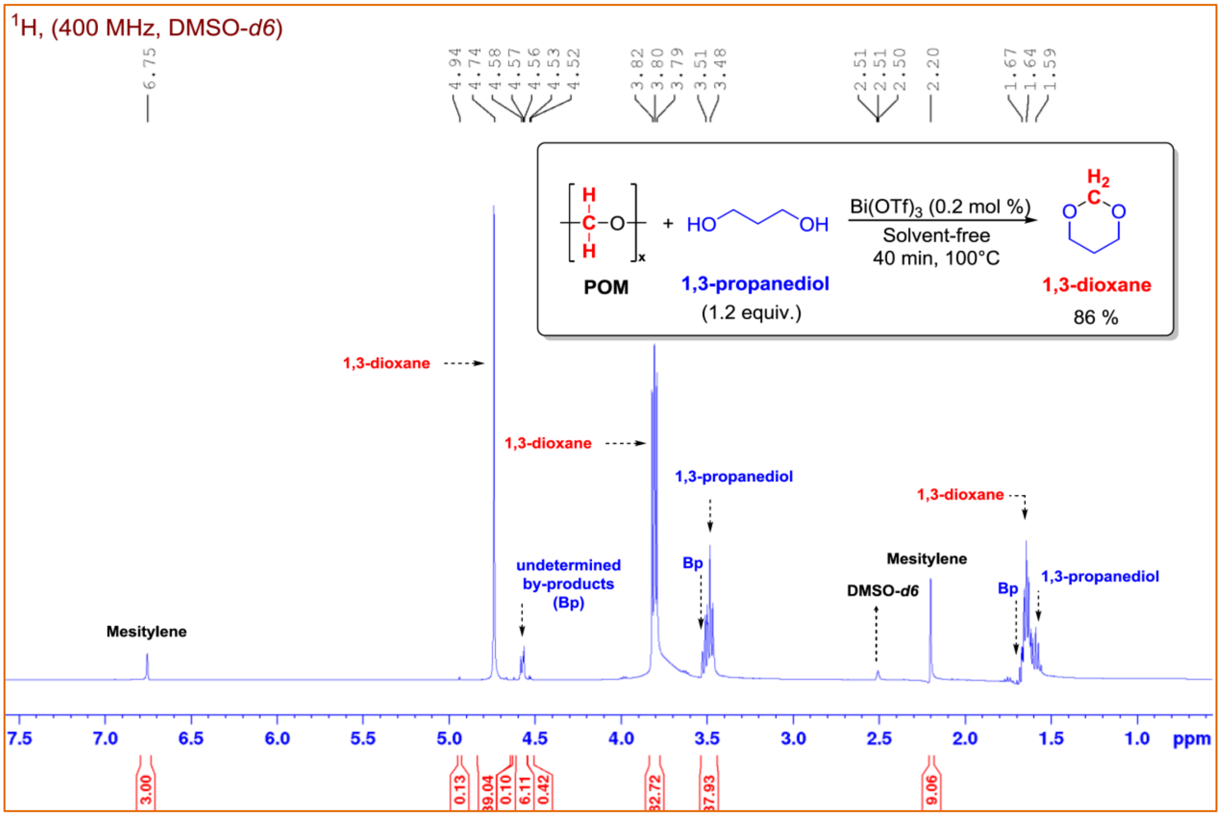

Fig. S6. ^{1}H-NMR spectrum (400 MHz) of the crude reaction mixture of the acetalization of 1,3-propanediol using Polyoxymethyelene homopolymer POM under neat conditions.

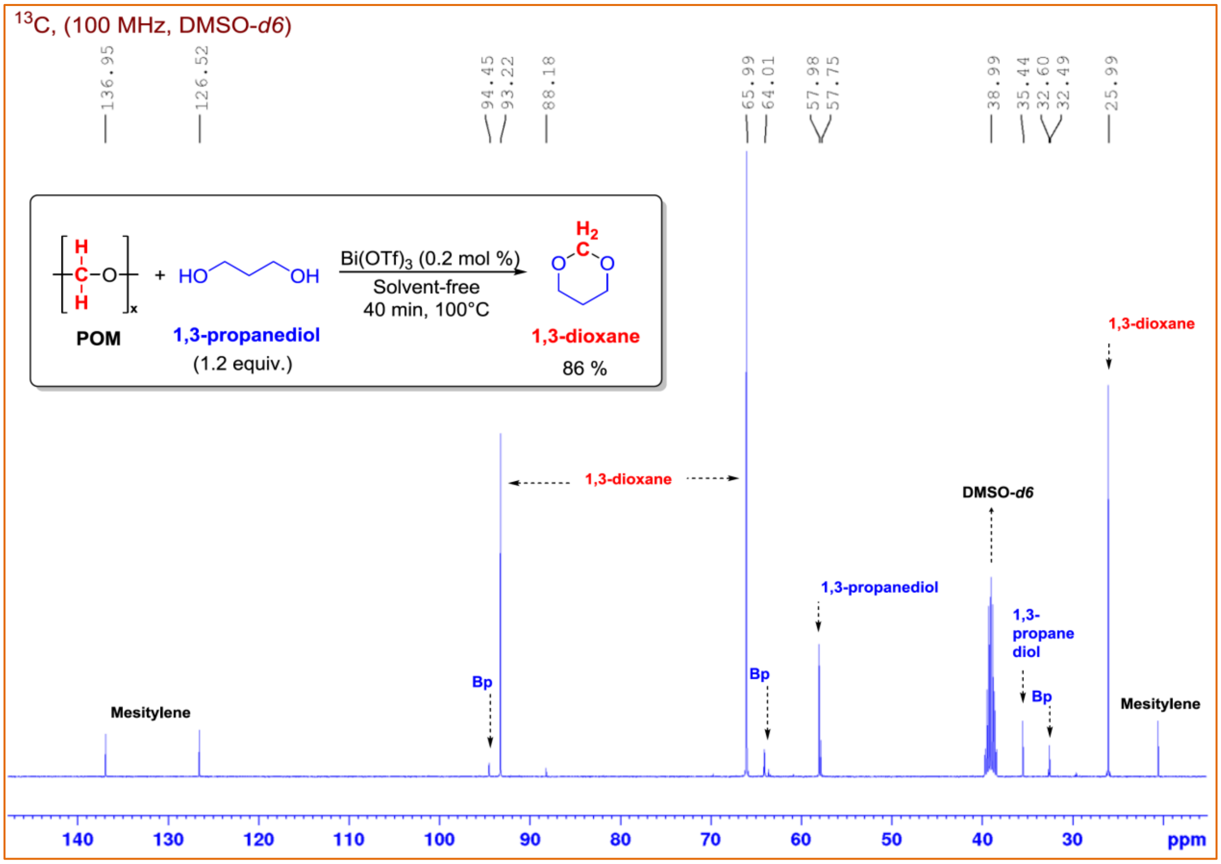

Fig. S7. **^{13}C-NMR spectrum (100 MHz) of the crude reaction mixture of the acetalization of 1,3-propanediol using Polyoxymethyelene homopolymer POM under neat conditions.**

NMR-Spectra of selected reactions towards the synthesis of cyclic acetals using diols and polyoxymethylene POM.

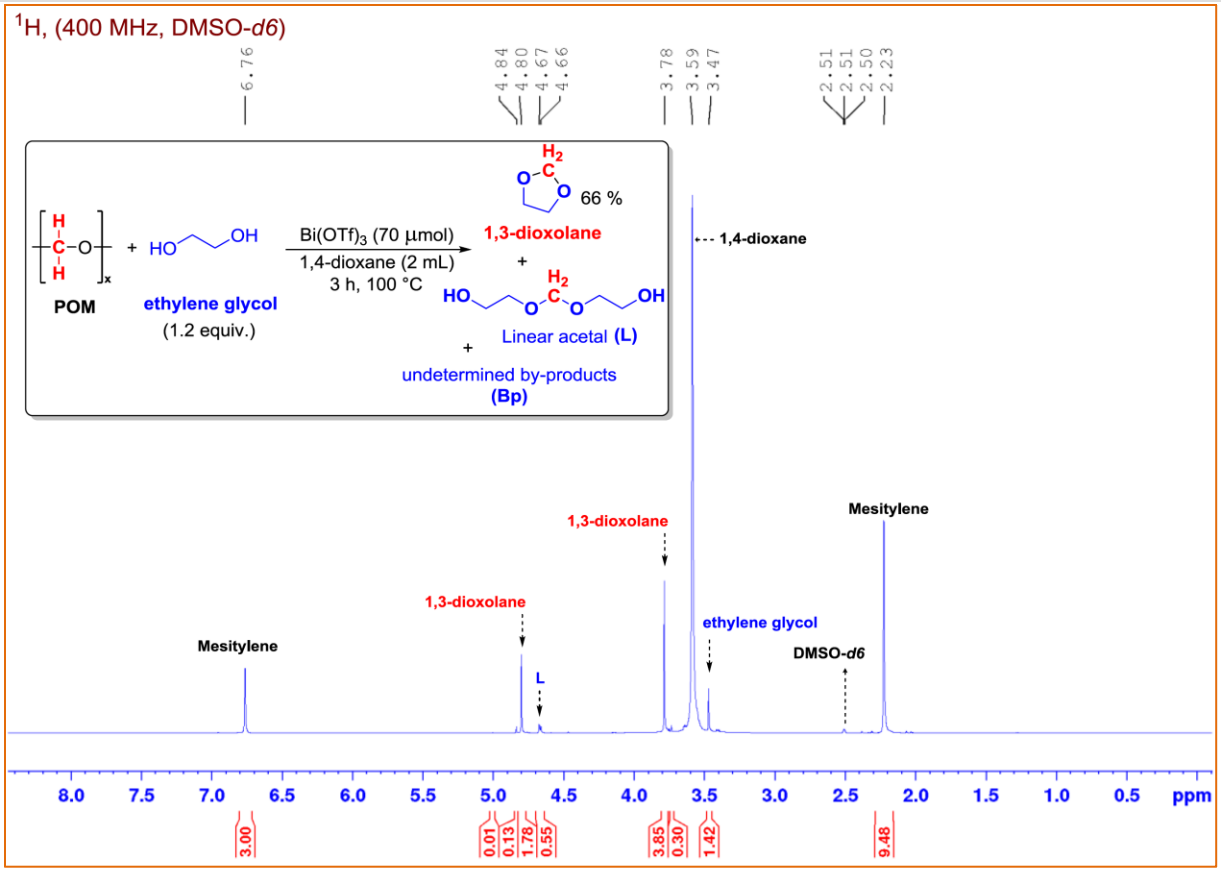

Fig. S8. **^{1}H-NMR spectrum (400 MHz) of the crude 1,4-dioxane reaction mixture of the acetalization of ethyleneglycol using Polyoxymethyelene homopolymer POM.**

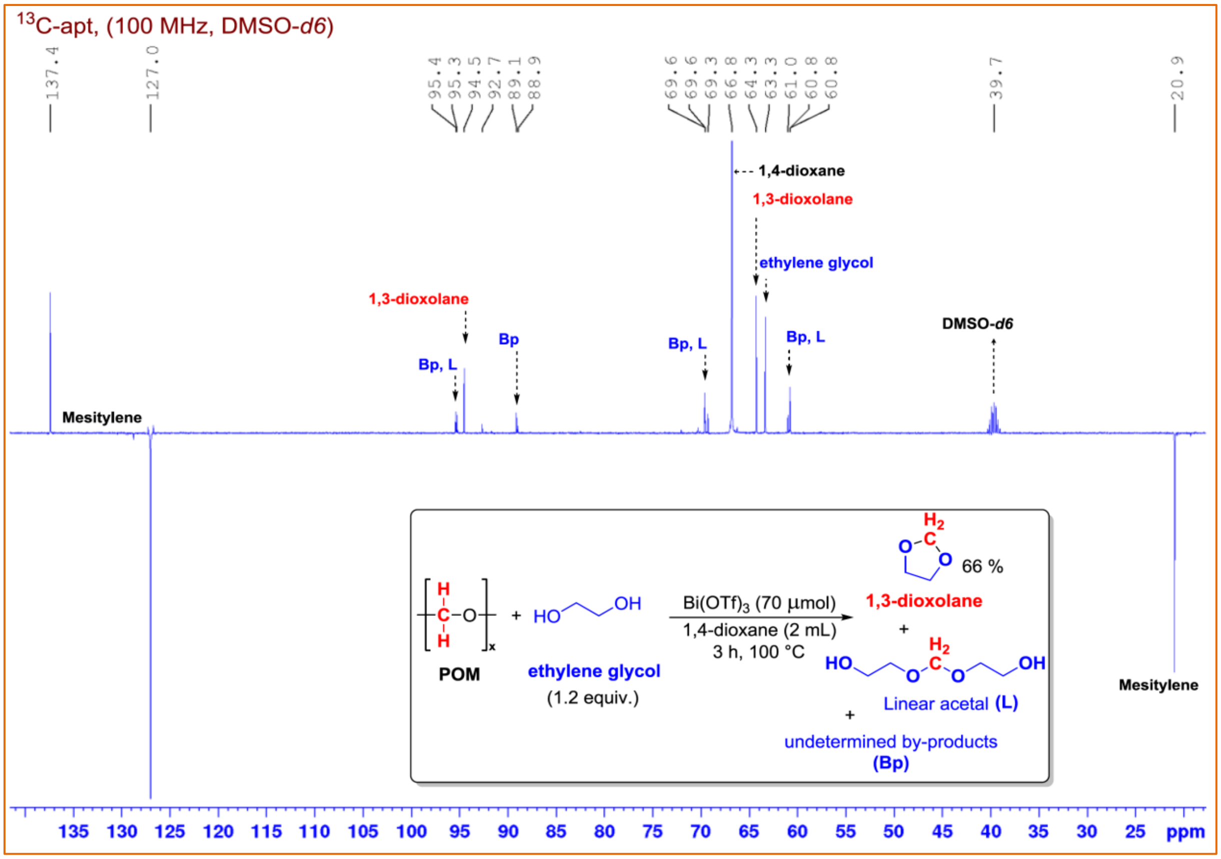

Fig. S9. ^{13}**C-NMR spectrum (100 MHz) of the crude 1,4-dioxane reaction mixture of the acetalization of ethyleneglycol using Polyoxymethyelene homopolymer POM.**

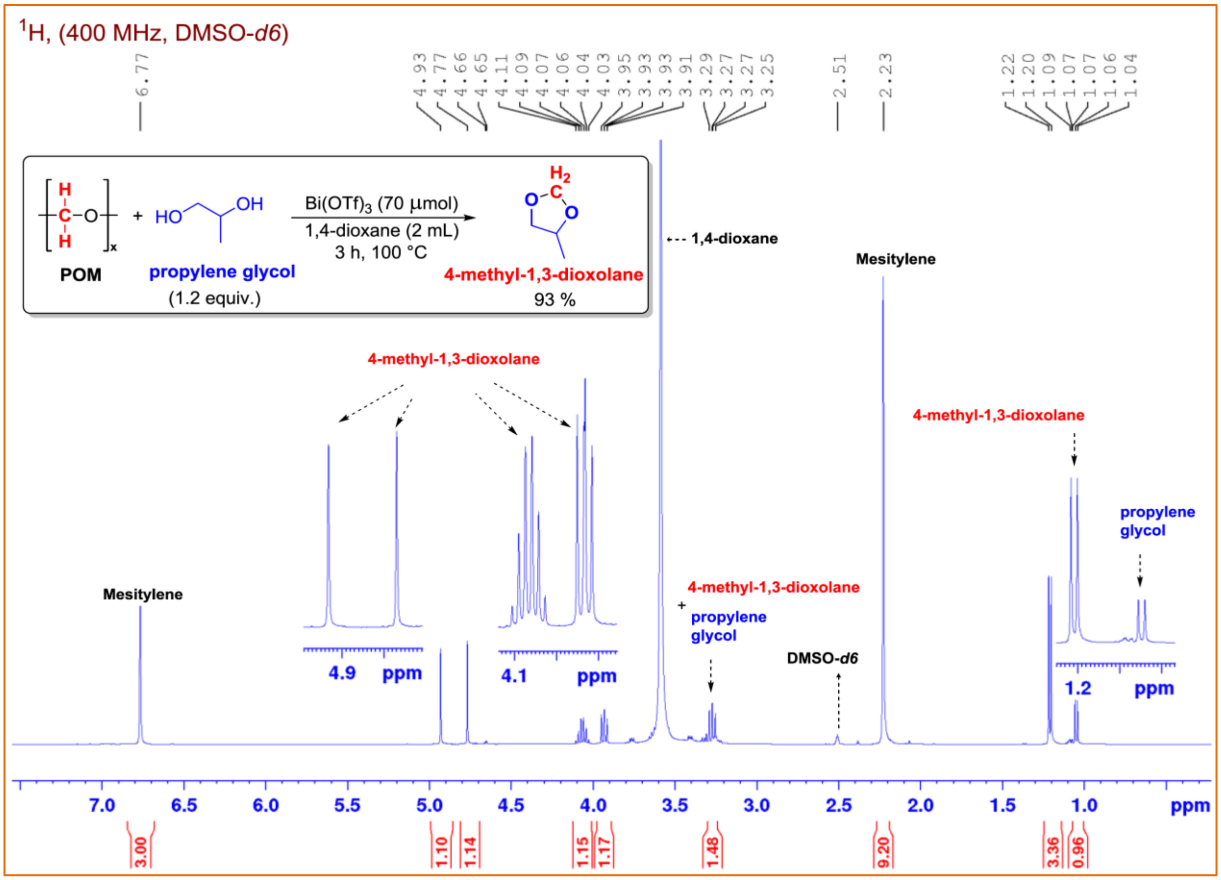

Fig. S10. **^{1}H-NMR spectrum (400 MHz) of the crude 1,4-dioxane reaction mixture of the acetalization of propyleneglycol using Polyoxymethyelene homopolymer POM.**

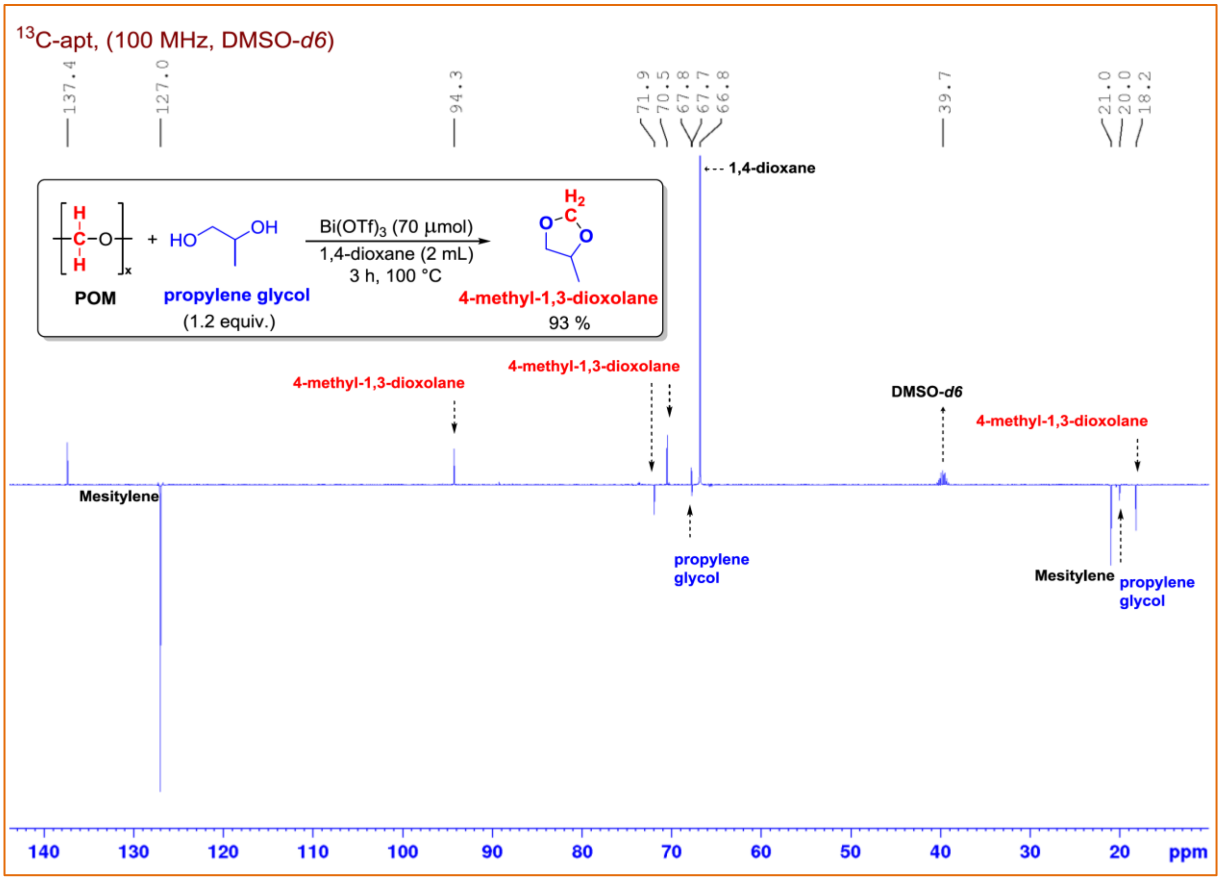

Fig. S11. **^{13}C-NMR spectrum (100 MHz) of the crude 1,4-dioxane reaction mixture of the acetalization of propyleneglycol using Polyoxymethyelene homopolymer POM.**

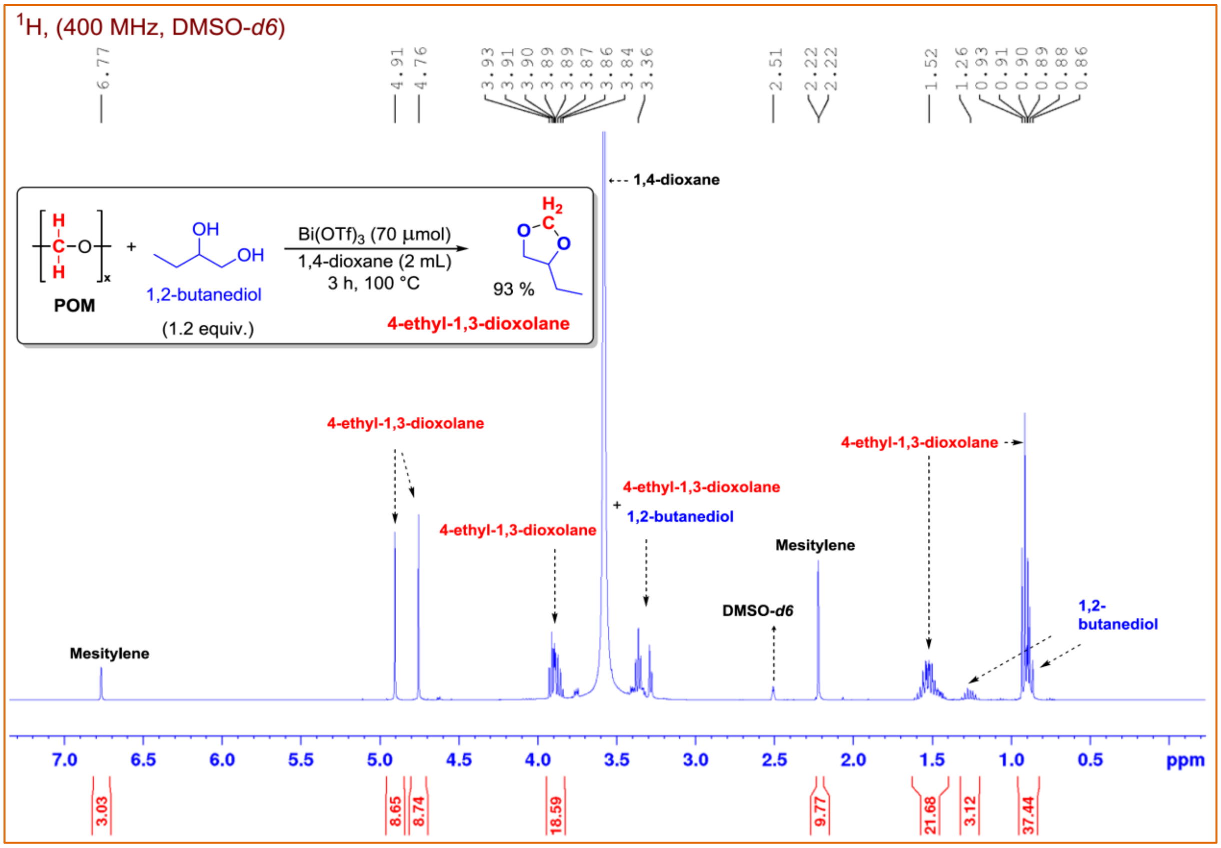

Fig. S12. ¹H-NMR spectrum (400 MHz) of the crude 1,4-dioxane reaction mixture of the acetalization of 1,2-butanediol using Polyoxymethyelene homopolymer POM.

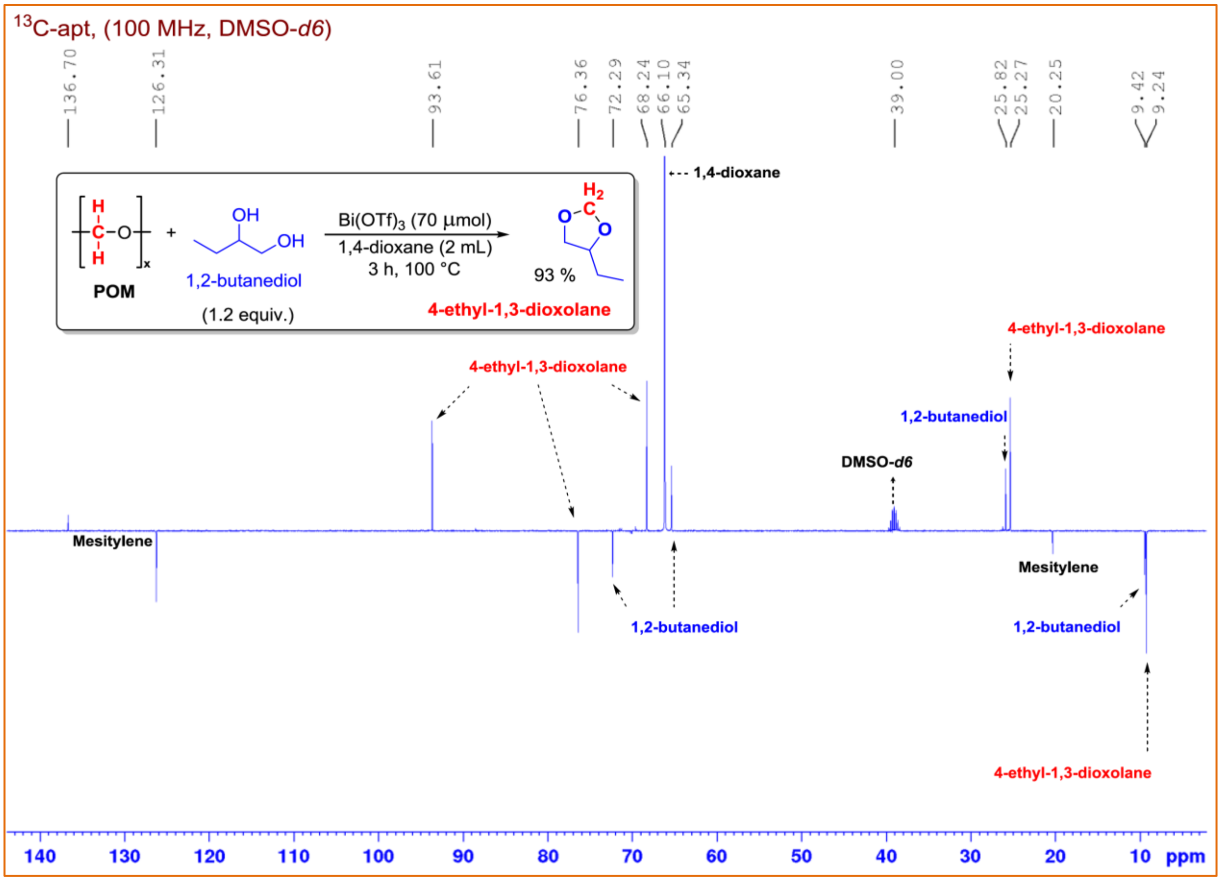

Fig. S13. **^{13}C-NMR spectrum (100 MHz) of the crude 1,4-dioxane reaction mixture of the acetalization of 1,2-butanediol using Polyoxymethyelene homopolymer POM.**

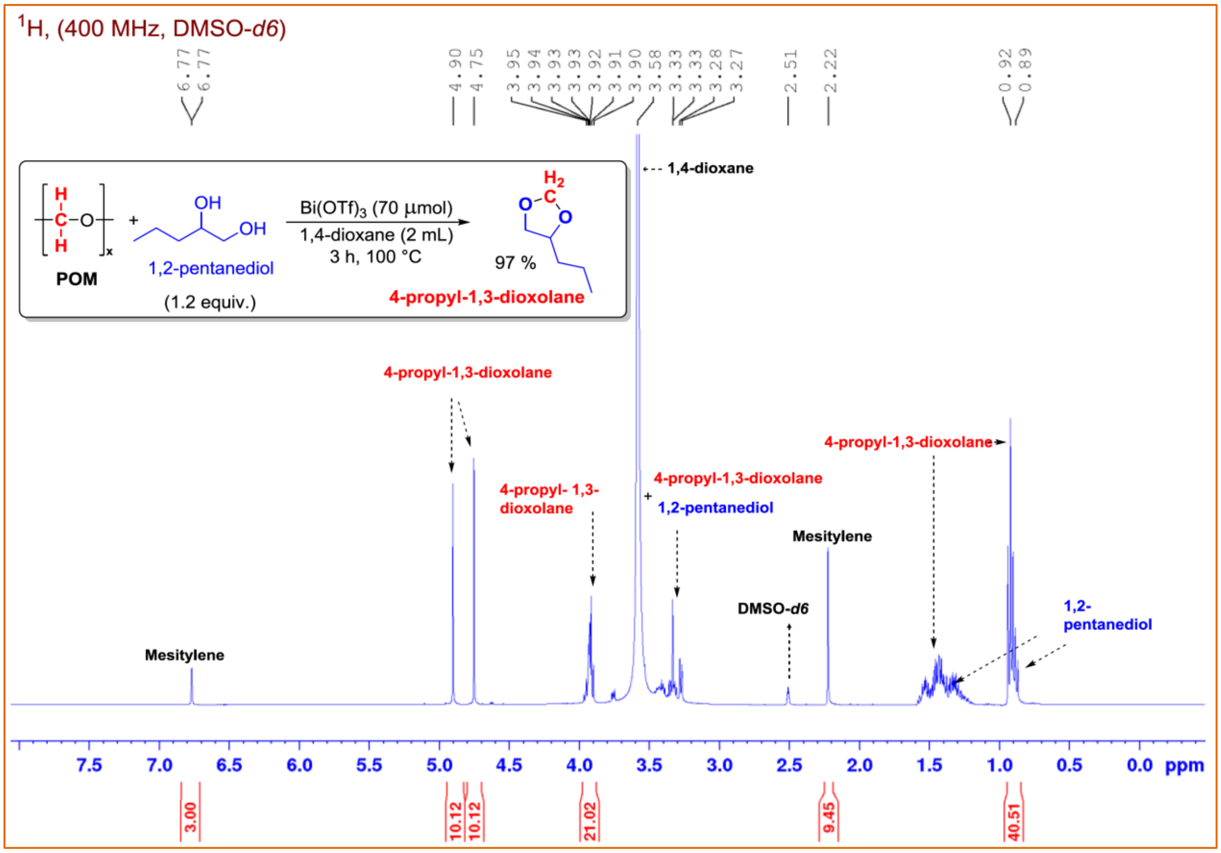

Fig. S14. ^{1}**H-NMR spectrum (400 MHz) of the crude 1,4-dioxane reaction mixture of the acetalization of 1,2-pentanediol using Polyoxymethyelene homopolymer POM.**

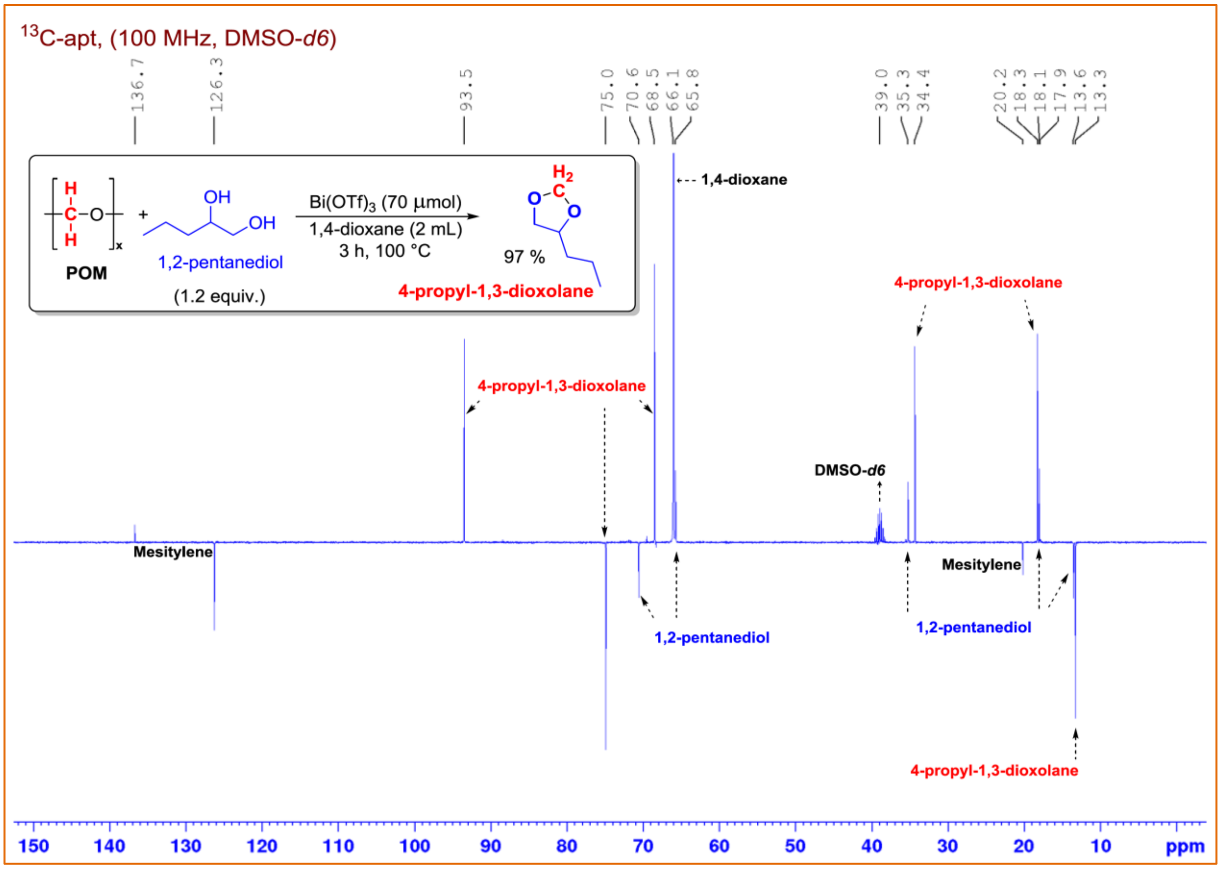

Fig. S15. **^{13}C-NMR spectrum (100 MHz) of the crude 1,4-dioxane reaction mixture of the acetalization of 1,2-pentanediol using Polyoxymethyelene homopolymer POM.**

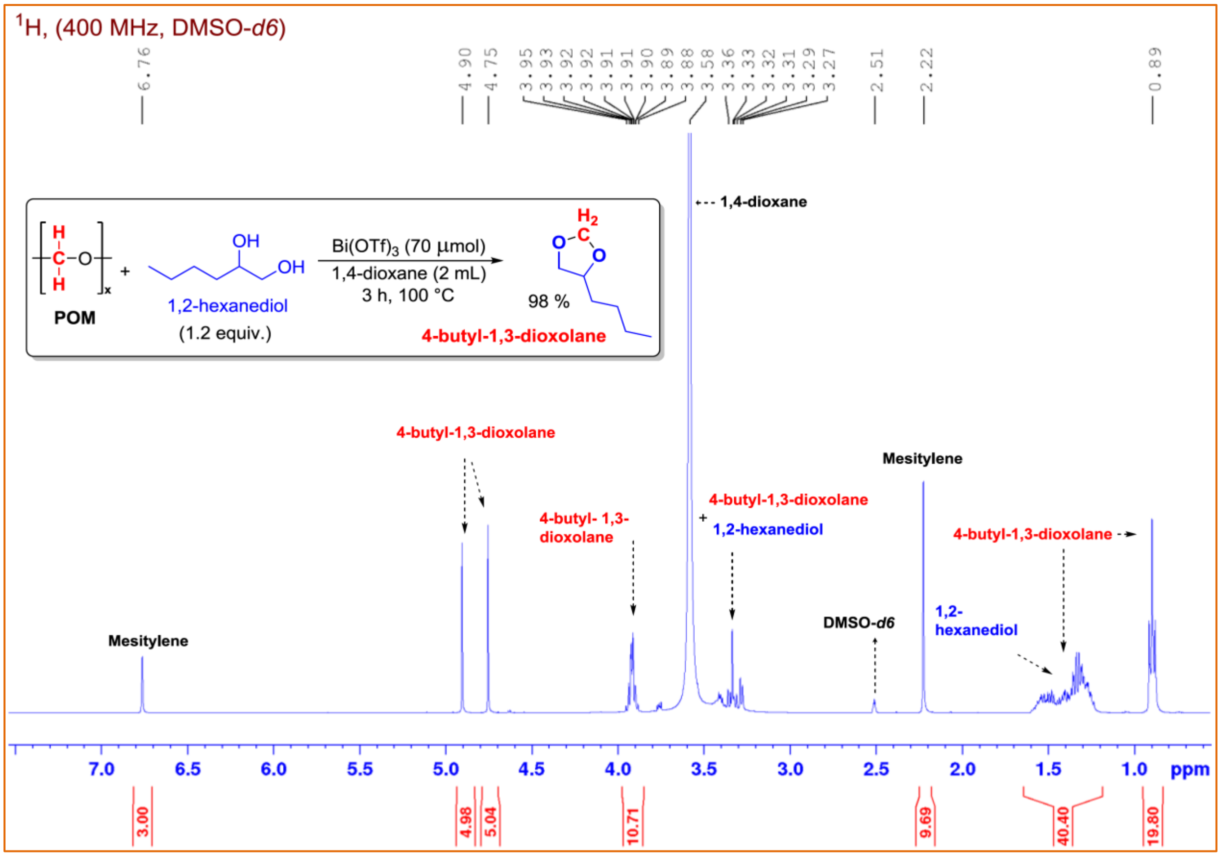

Fig. S16. **^{1}H-NMR spectrum (400 MHz) of the crude 1,4-dioxane reaction mixture of the acetalization of 1,2-hexanediol using Polyoxymethyelene homopolymer POM.**

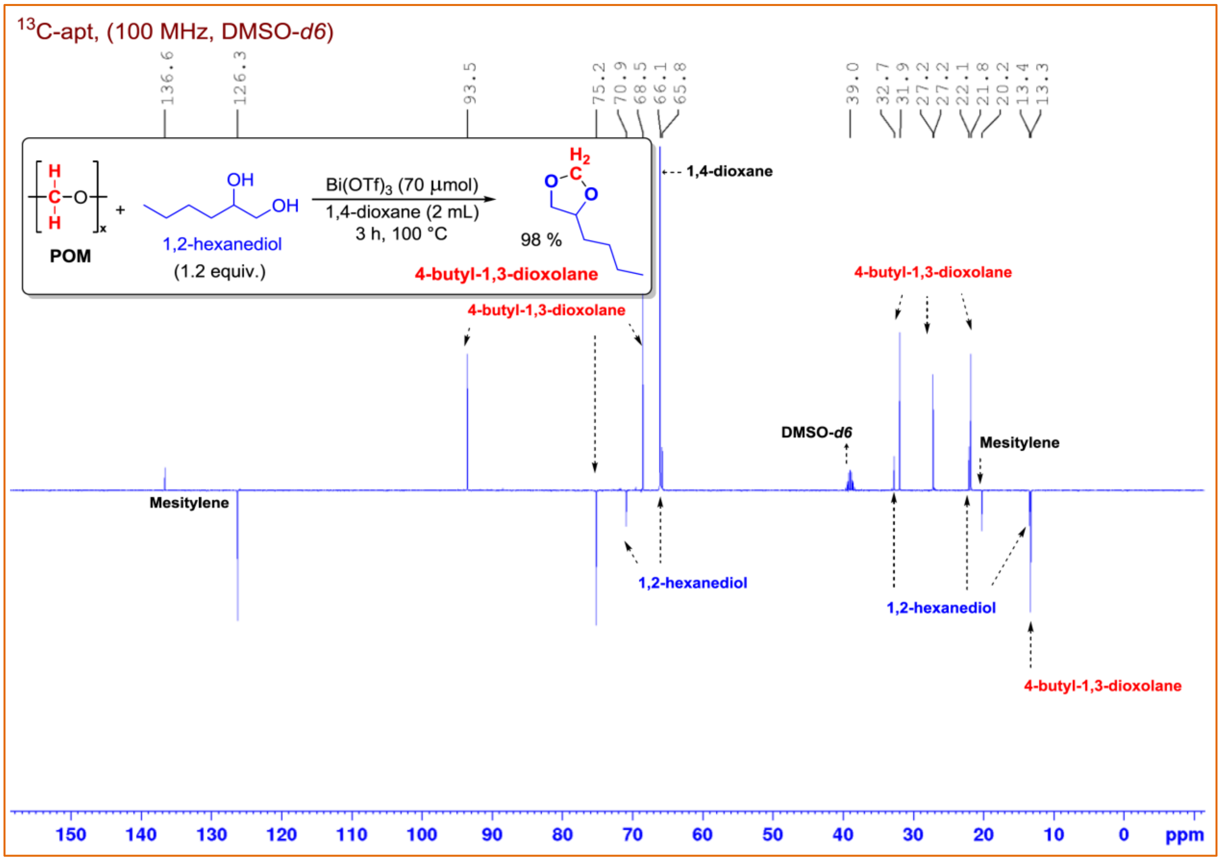

Fig. S17. ^{13}**C-NMR spectrum (100 MHz) of the crude 1,4-dioxane reaction mixture of the acetalization of 1,2-hexanediol using Polyoxymethyelene homopolymer POM.**

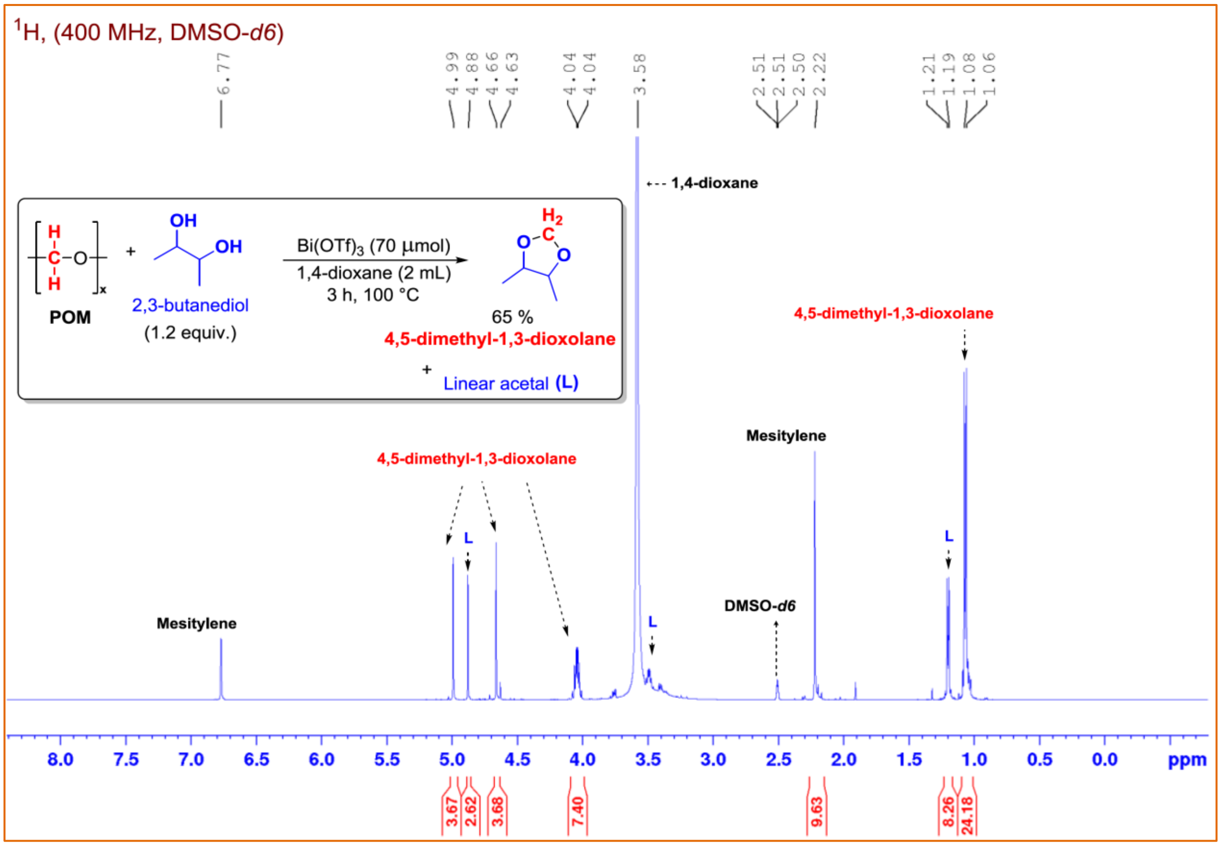

Fig. S18. ^{1}**H-NMR spectrum (400 MHz) of the crude 1,4-dioxane reaction mixture of the acetalization of 2,3-butanediol using Polyoxymethyelene homopolymer POM.**

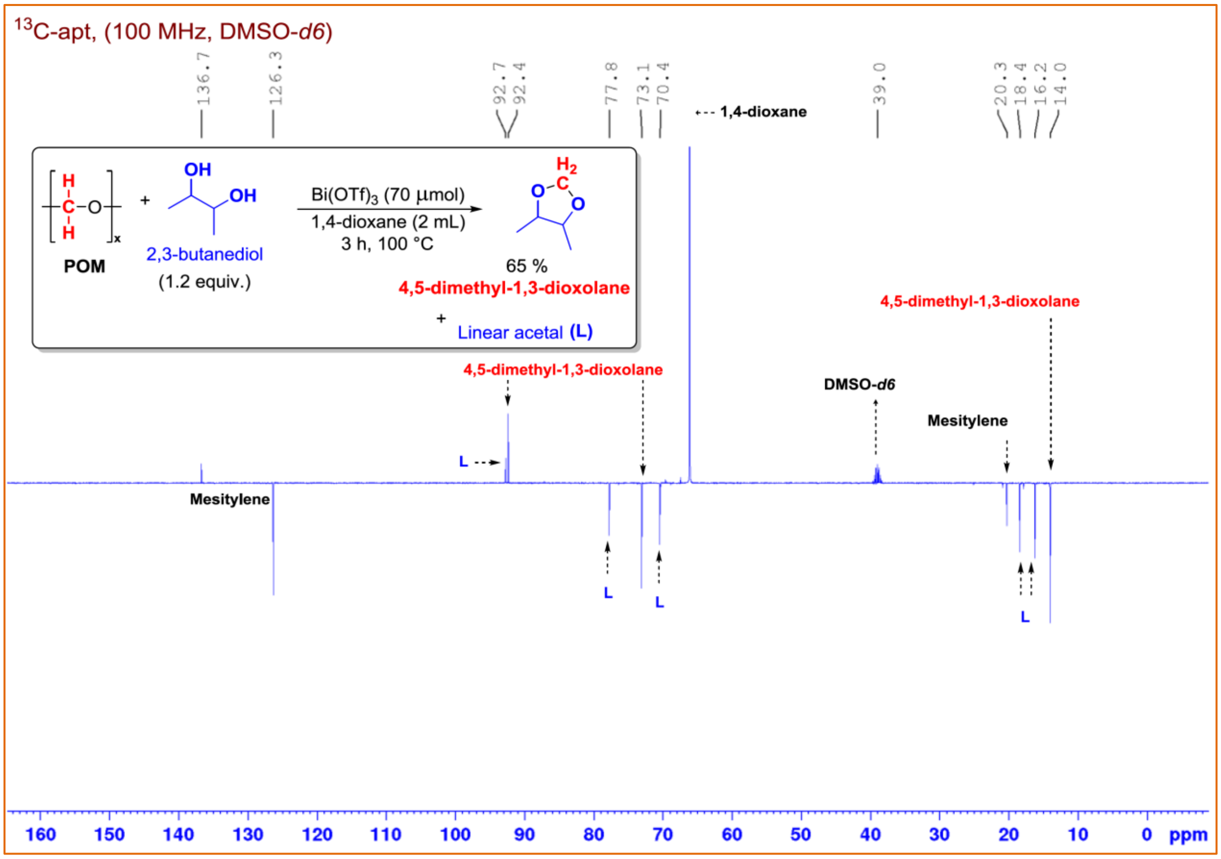

Fig. S19. ^{13}**C-NMR spectrum (100 MHz) of the crude 1,4-dioxane reaction mixture of the acetalization of 2,3-butanediol using Polyoxymethyelene homopolymer POM.**

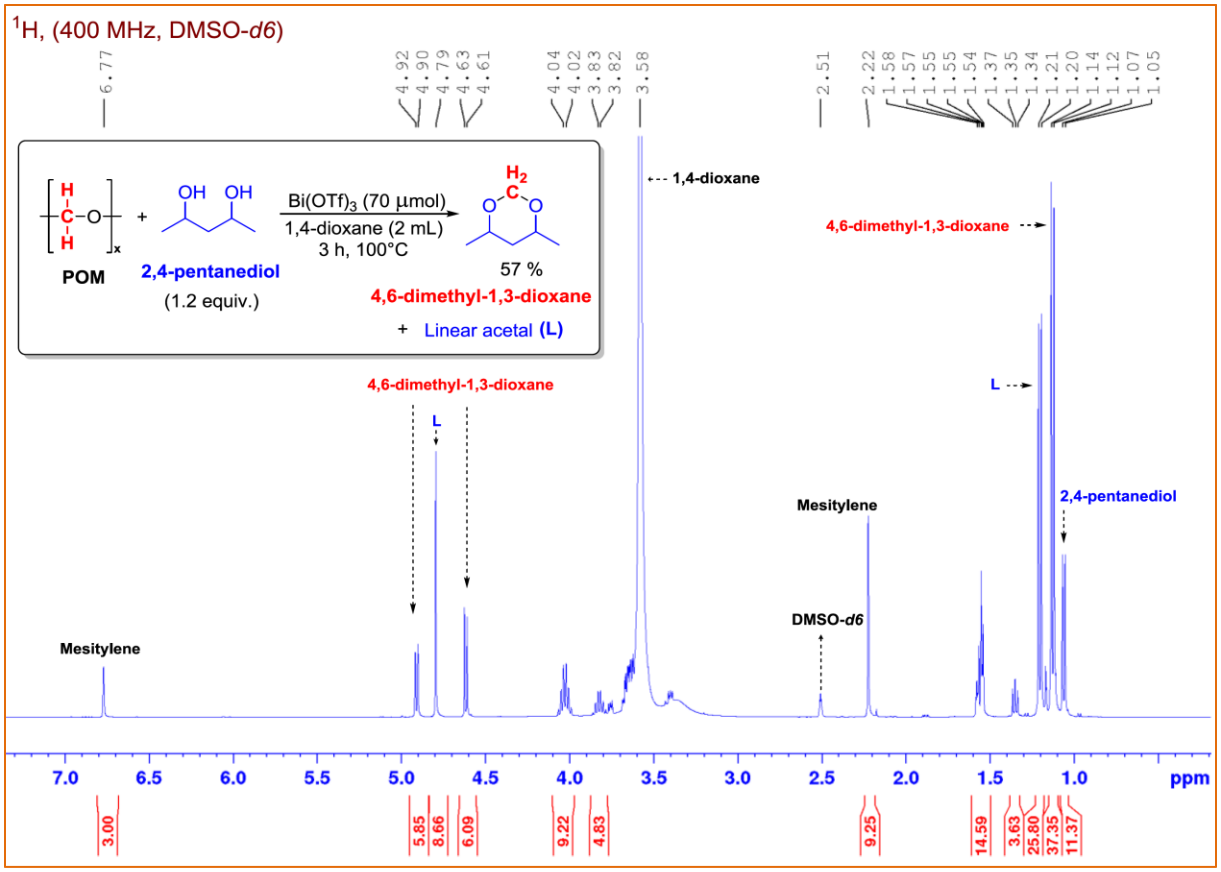

Fig. S20. **^{1}H-NMR spectrum (400 MHz) of the crude 1,4-dioxane reaction mixture of the acetalization of 2,4-pentanediol using Polyoxymethyelene homopolymer POM.**

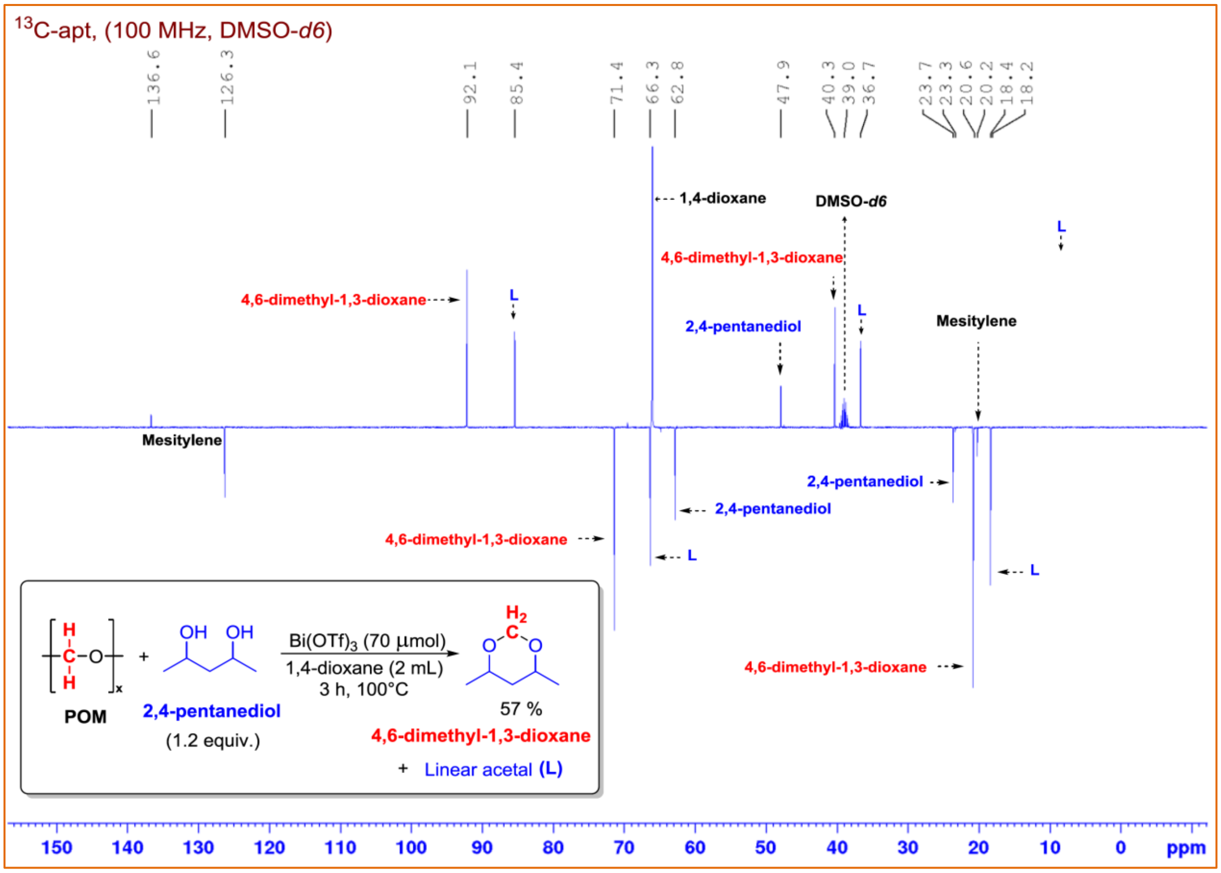

Fig. S21. ^{13}C-NMR spectrum (100 MHz) of the crude 1,4-dioxane reaction mixture of the acetalization of 2,4-pentanediol using Polyoxymethyelene homopolymer POM.

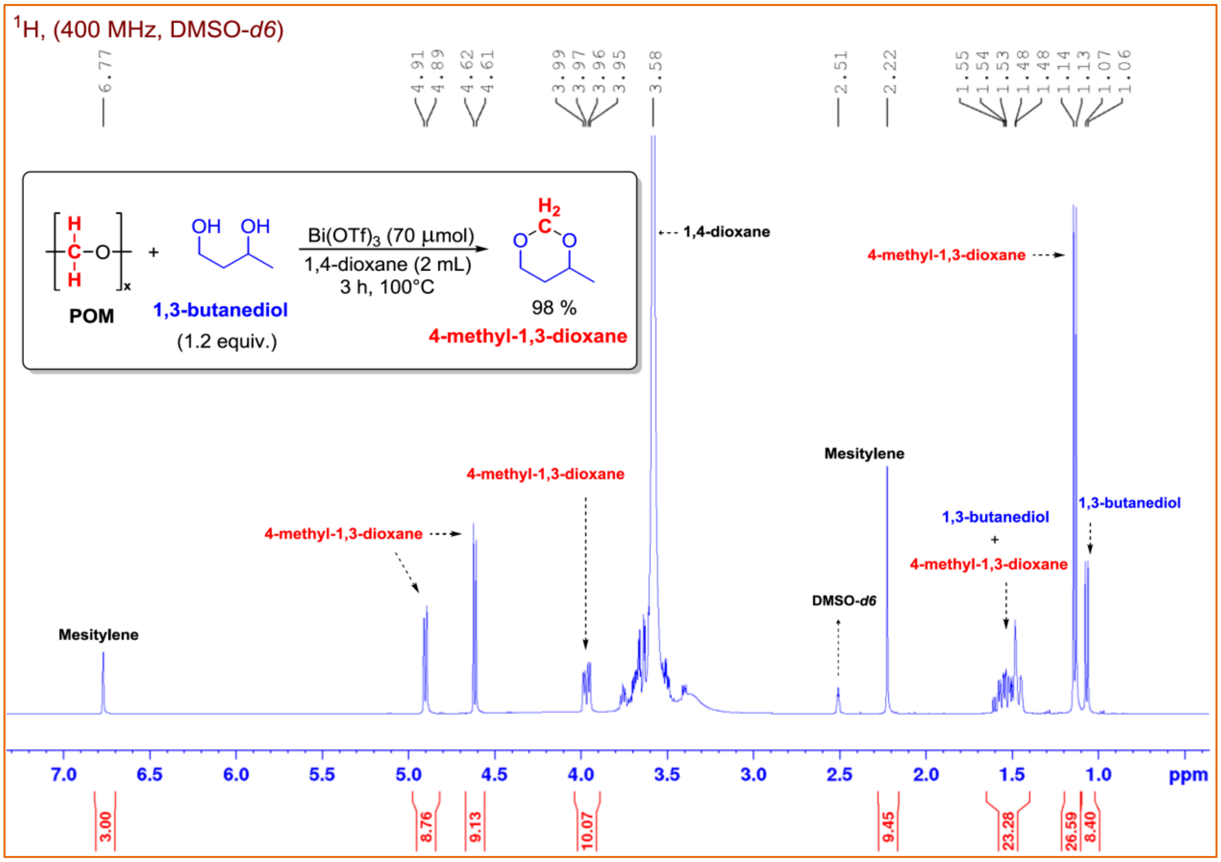

Fig. S22. ^{1}**H-NMR spectrum (400 MHz) of the crude 1,4-dioxane reaction mixture of the acetalization of 1,3-butanediol using Polyoxymethyelene homopolymer POM.**

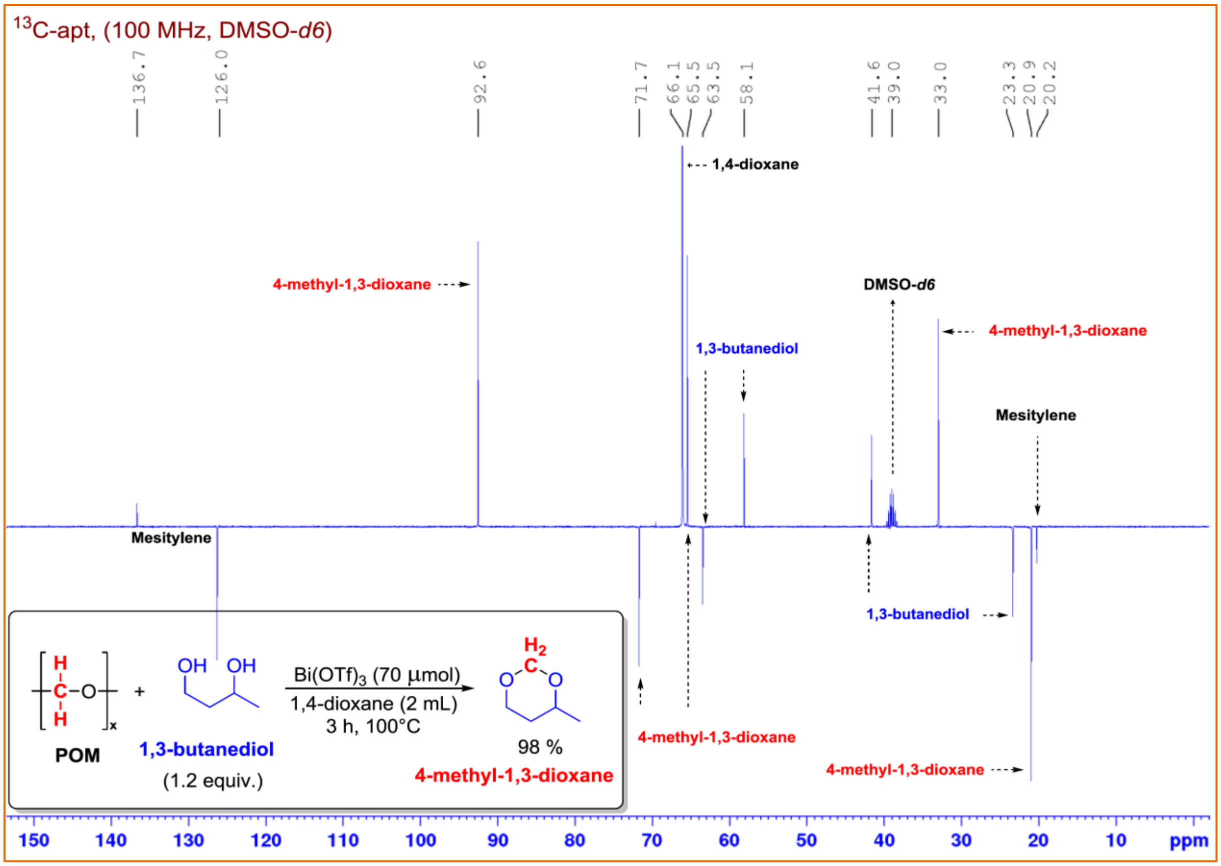

Fig. S23. ^{13}**C-NMR spectrum (100 MHz) of the crude 1,4-dioxane reaction mixture of the acetalization of 1,3-butanediol using Polyoxymethyelene homopolymer POM.**

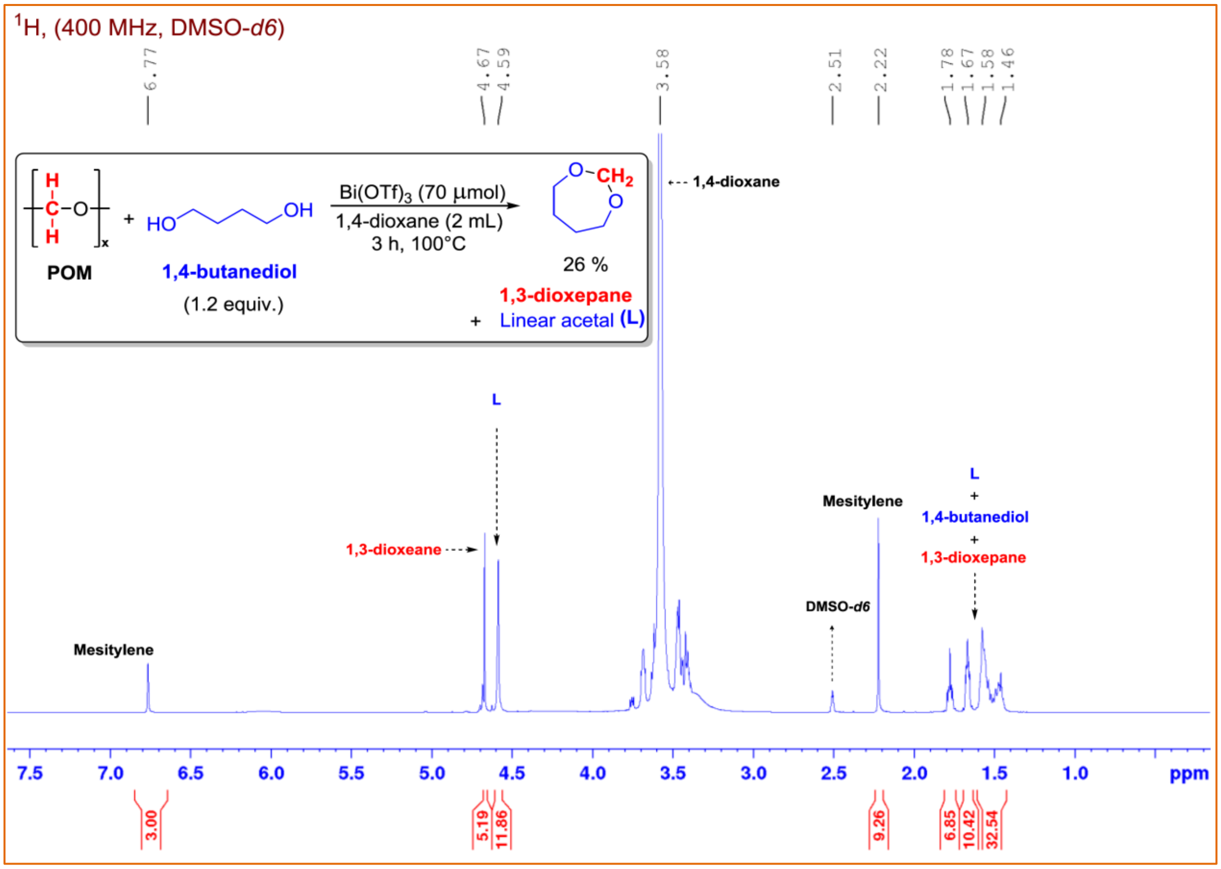

Fig. S24. **^{1}H-NMR spectrum (400 MHz) of the crude 1,4-dioxane reaction mixture of the acetalization of 1,4-butanediol using Polyoxymethylene homopolymer POM.**

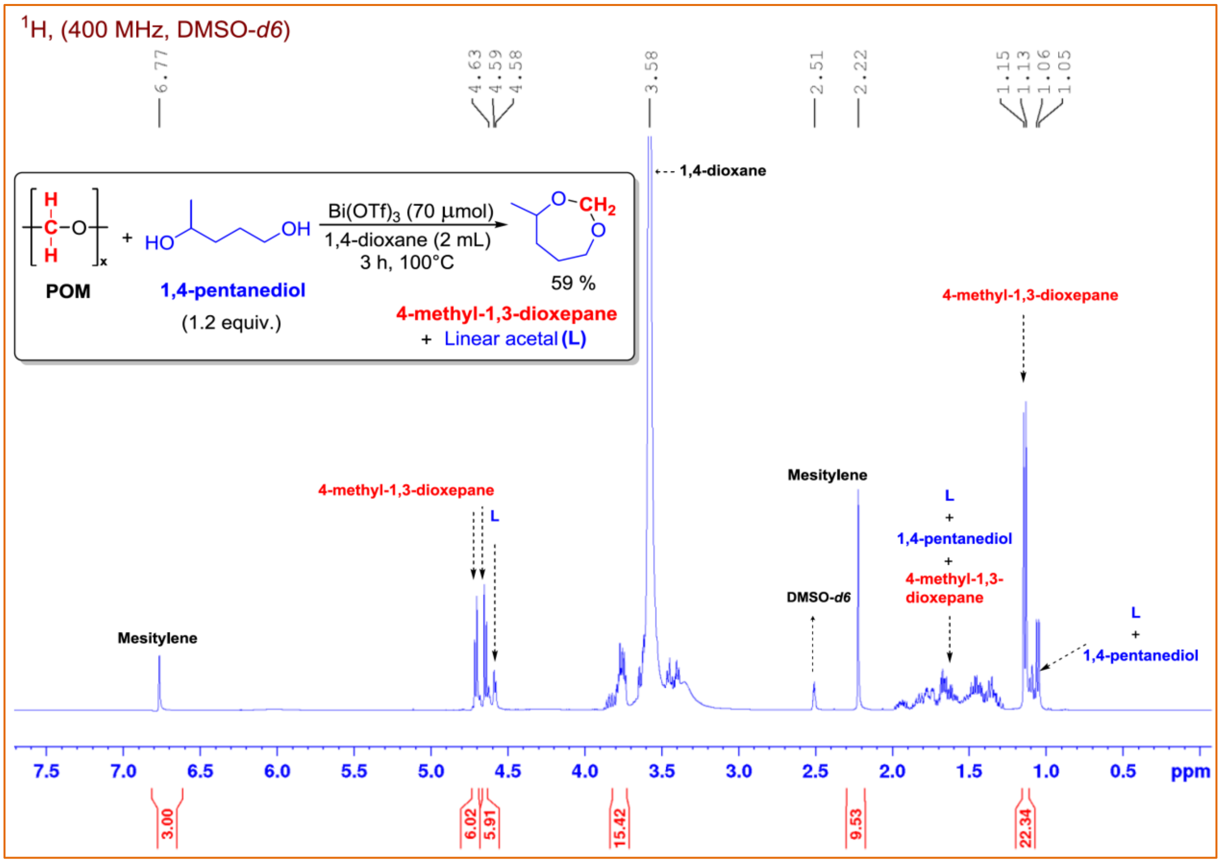

Fig. S25. ^{1}**H-NMR spectrum (400 MHz) of the crude 1,4-dioxane reaction mixture of the acetalization of 1,4-pentanediol using Polyoxymethyelene homopolymer POM.**

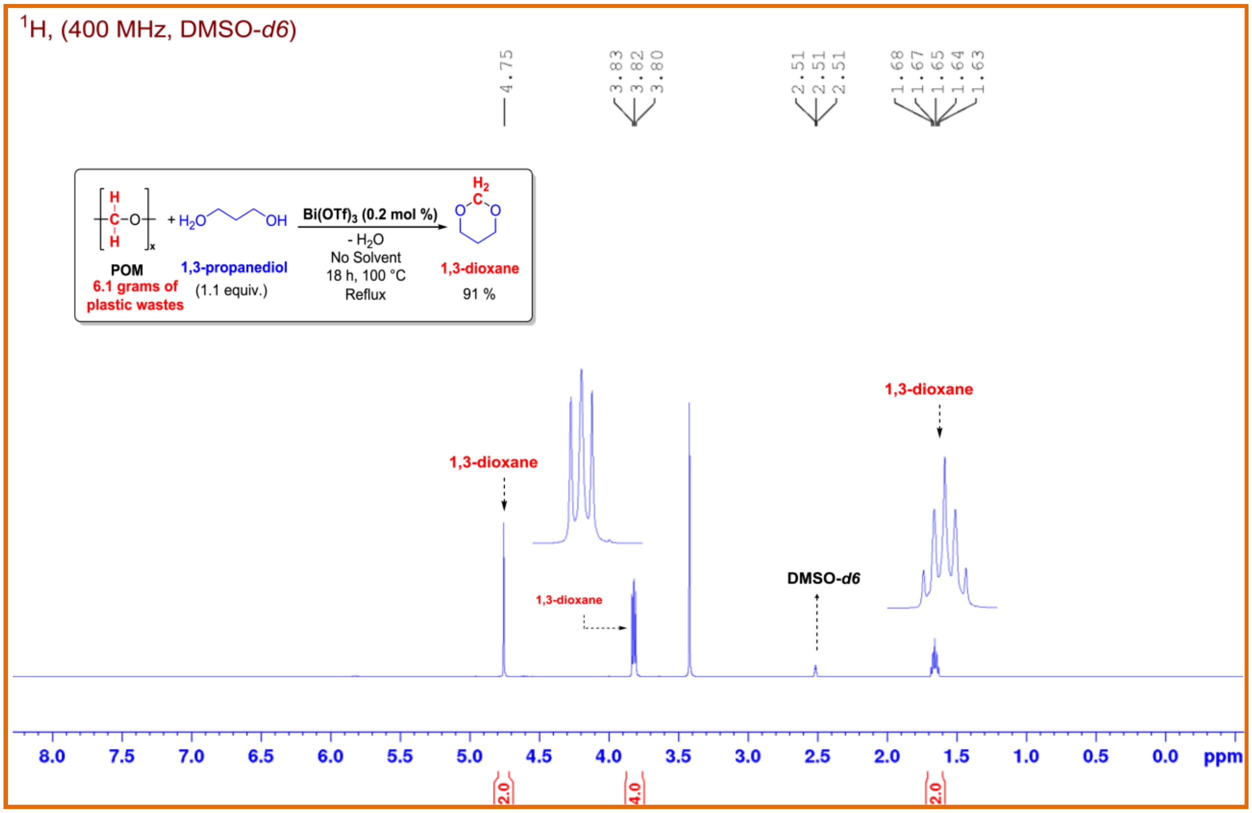

Fig. S26. **^{1}H-NMR spectrum (400 MHz) of the pure 1,3-dioxane product afforded by the solvent-free scale-up reaction of POM waste plastic mixture and 1,3-propanediol.**

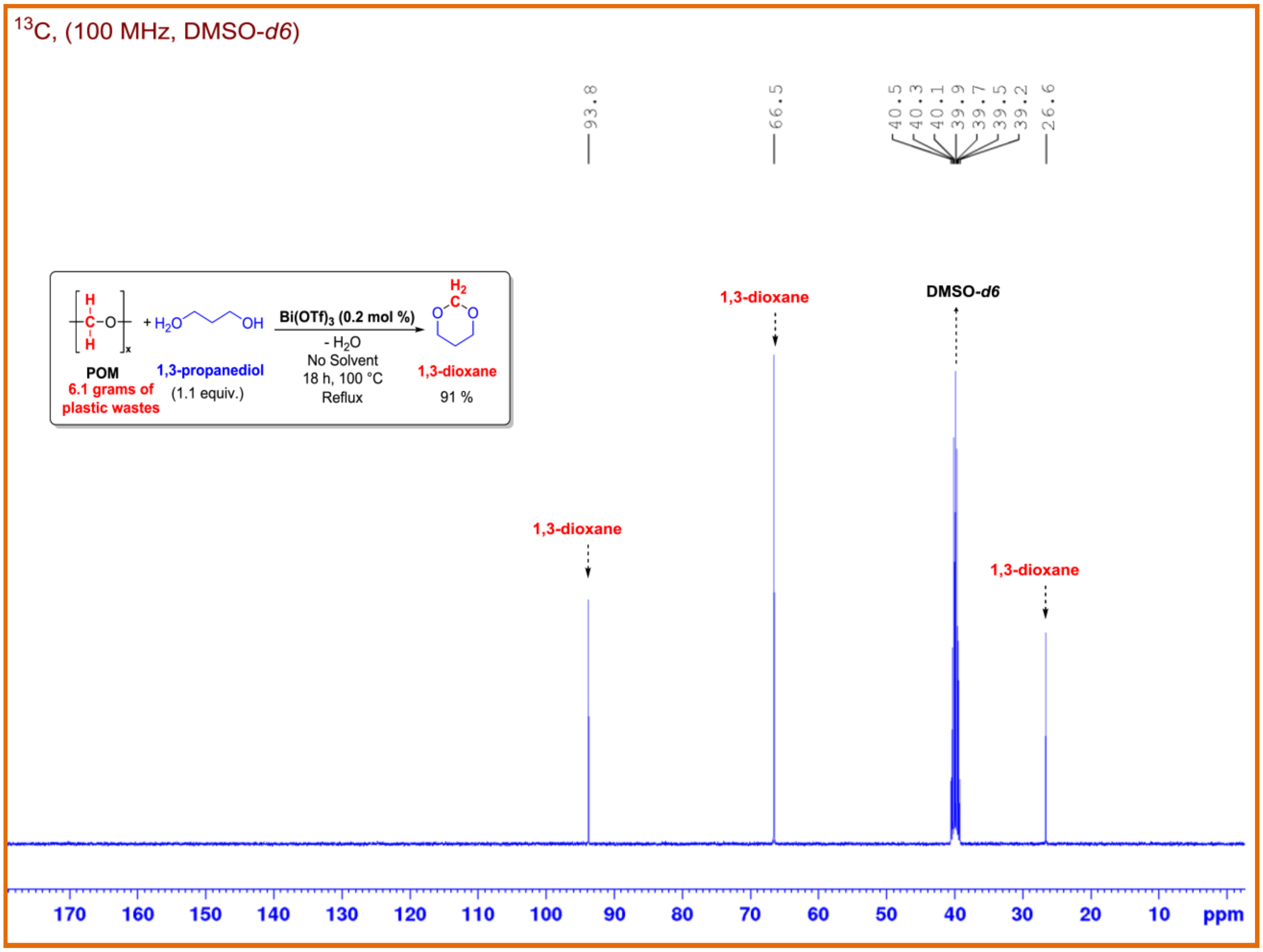

Fig. S27. ^{13}**C-NMR spectrum (100 MHz) of the pure 1,3-dioxane product afforded by the solvent-free scale-up reaction of POM waste plastic mixture and 1,3-propanediol.**

[1] S. D. Nogare, J. O. Punderson, E.I. Du Pont De Nemours & Co., US2998409, USA, **1961**.
